# Multivariate random forest prediction of poverty and malnutrition prevalence

**DOI:** 10.1371/journal.pone.0255519

**Published:** 2021-09-08

**Authors:** Chris Browne, David S. Matteson, Linden McBride, Leiqiu Hu, Yanyan Liu, Ying Sun, Jiaming Wen, Christopher B. Barrett

**Affiliations:** 1 Center for Applied Mathematics, Cornell University, Ithaca, NY, United States of America; 2 Department of Statistics & Data Science, Cornell University, Ithaca, NY, United States of America; 3 Department of Economics, St. Mary’s College of Maryland, St. Mary’s City, MD, United States of America; 4 Department of Atmospheric and Earth Science, University of Alabama in Huntsville, Huntsville, AL, United States of America; 5 Markets, Trade and Institutions Division, International Food Policy Research Institute, Washington, DC, United States of America; 6 School of Integrative Plant Science, Soil and Crop Sciences Section, Cornell University, Ithaca, NY, United States of America; 7 Charles H. Dyson School of Applied Economics and Management, Cornell University, Ithaca, NY, United States of America; United Nations University Institute for Natural Resources in Africa, GHANA

## Abstract

Advances in remote sensing and machine learning enable increasingly accurate, inexpensive, and timely estimation of poverty and malnutrition indicators to guide development and humanitarian agencies’ programming. However, state of the art models often rely on proprietary data and/or deep or transfer learning methods whose underlying mechanics may be challenging to interpret. We demonstrate how interpretable random forest models can produce estimates of a set of (potentially correlated) malnutrition and poverty prevalence measures using free, open access, regularly updated, georeferenced data. We demonstrate two use cases: contemporaneous prediction, which might be used for poverty mapping, geographic targeting, or monitoring and evaluation tasks, and a sequential nowcasting task that can inform early warning systems. Applied to data from 11 low and lower-middle income countries, we find predictive accuracy broadly comparable for both tasks to prior studies that use proprietary data and/or deep or transfer learning methods.

## 1 Introduction

Governments and humanitarian agencies devote considerable resources towards poverty and malnutrition reduction efforts. One key factor in the effectiveness of such efforts is the accuracy with which poor and malnourished populations can be identified. Accurate identification of poor or malnourished populations in space and time serves multiple purposes [1]. Nowcasting—i.e., using current observations of predictive features combined with past observations of the poverty or malnutrition outcome(s) of interest—can help with geographic needs assessments and targeting, as well as provide baseline measures for impact evaluation of interventions. Contemporaneous prediction—i.e., estimation of locations not covered in standard household surveys—can fill in the gaps in survey evidence, generating poverty maps for geographic targeting of interventions and to inform ongoing monitoring and evaluation activities. The more precise and interpretable the estimates, and the more parsimonious and inexpensive the data demands of the model, the greater the likelihood that agencies can employ such methods to accurately target and evaluate interventions to address agricultural, economic, political, or weather shocks that might otherwise thrust vulnerable groups into poverty traps or famine [2, 3].

To inform aid targeting, monitoring and evaluation efforts, agencies have historically drawn data mainly from detailed household surveys, such as the large-scale, nationally-representative Demographic and Healthy Surveys (DHS) or Living Standards Measurement Study (LSMS) programs. Such surveys are, however, expensive and time-consuming, and may systematically omit subregions that are harder or more dangerous to physically access, despite the severe poverty and malnutrition prevalence often endemic to such locations. Moreover, high-quality, large-scale surveys are typically fielded only once every several years, and are generally statistically representative of the population only at relatively large (e.g., provincial or regional) scales under standard sampling protocols. Although higher frequency surveys can improve the timeliness of survey coverage, this often comes at the cost of spatial and survey detail [4]. While enormously useful, the shortcomings of these survey-based estimation techniques for up to date or forward-looking poverty and malnutrition prevalence can ultimately hinder the development of timely and effective development and humanitarian programming, especially in circumstances where rapid response is needed.

Recent advances in remote sensing (RS) and machine learning (ML) offer tantalizing prospects to resolve some of these shortcomings by providing accurate, cheap, and timely indicators of poverty and malnutrition status, at high spatio-temporal resolution, and the means to translate these data into actionable predictions for policy. Most of the recent literature applying statistical and ML methods to predict poverty and malnutrition status focus on the contemporaneous prediction case, which bears important similarities to the well-established poverty mapping literature, which is in turn based on small area estimation methods [1, 5–7].

More recently, Jean *et al*. [8] pioneered the use of deep learning methods, and in particular convolutional neural networks trained in conjunction with transfer learning methods and RS data, to identify nationwide poverty incidence at high spatial resolution (i.e., village or county level). Their and subsequent innovations promise much lower data costs and more current estimates than conventional poverty mapping methods that rely on household surveys and census data alone. The past few years have brought many useful refinements of these methods, alongside variations in precise statistical methodology and target outcome [9–18].

Although famine early warning system developers have over the last decade made heavy use of RS data measuring weather patterns and vegetation health alongside manually collected food price data as key information sources in qualitative models [19, 20], they have made limited use of statistical models to forecast future or even nowcast current poverty or malnutrition status. Exceptions to this general rule include Mude *et al*. [21], Yeh *et al*. [13], and Tang *et al*. [22]. Mude *et al*. [21] use several years of monthly anthropometric and remotely sensed vegetative and climate data to predict children’s mid-upper arm circumference in northern Kenya at the community level (roughly equivalent to DHS survey clusters) using multivariate regression methods, while Yeh *et al*. [13] and Tang *et al*. [22], apply deep learning methods to predict temporal changes in household poverty measures using multispectral satellite imagery and RS vegetative indices respectively.

Ongoing challenges in this fast-moving field include the development of rigorous methods that can produce accurate and timely predictions of poverty and malnutrition status using open access and near-real time data, to assess how the quality of these predictions change when moving between contemporaneous prediction and forecasting/nowcasting, and to identify the types of RS data that are most valuable for such predictions. This paper engages with these challenges by demonstrating how free, open access, regularly updated, georeferenced data can be analyzed using interpretable random forest models to provide estimates of a set of malnutrition and poverty indicators, with accuracy broadly comparable (in some cases, perhaps superior) to estimates based on deep learning methods.

We demonstrate this for two use cases: a) contemporaneous mapping of poverty and malnutrition indicators, i.e., contemporaneously predicting prevalence at both surveyed and unsurveyed locations within a given country and survey year, and b) early warning, or nowcasting near-future prevalence levels based on historical observations and current RS data. Consistent with prior findings, we find it is much easier to predict the prevalence of asset poverty than of child malnutrition indicators [10], and find early warning to be more challenging than contemporaneous prediction [13]. Because poverty and malnutrition indicators are typically correlated, we also examine whether multivariate methods, which predict several indicators simultaneously, could enhance poverty and malnutrition prediction, and find mixed results. In some but not all cases, the accuracy of predictions of multiple indicators modestly exceeds that of predicting each independently.

In explaining poverty and malnutrition prevalence, we find that geographic variables that are unlikely to change much over just a few years have the greatest explanatory power, followed by vegetation and climate data. Although conflict and food price shocks elicit considerable attention, we find they contribute relatively little to the prediction of malnutrition and poverty indicators. As a final contribution, we also provide our full set of training and testing data in the hopes that its availability might further global efforts in poverty and malnutrition prediction.

## 2 Data description

We focus on data from eleven USAID Feed the Future (FTF) priority countries: Bangladesh, Ethiopia, Ghana, Guatemala, Honduras, Kenya, Mali, Nepal, Nigeria, Senegal, and Uganda. In feature selection, we restrict ourselves to publicly available data so as to represent what might be feasible for agencies unable to invest scarce resources in data collection or procurement. We note that although publicly available data have become increasingly plentiful, curation of standardized, spatially and temporally matched, regularly updated data remains an undersupplied public good. In this section we describe our data sources before discussing pre-processing in the next section. Links to all data sources considered in this work can be found in S5 Table in S1 File, while the processed data used in this work is available for download at http://barrett.dyson.cornell.edu/files/research/data.csv. This data has also been attached as supplemental information accompanying this manuscript. We inform our choice of features based on variables shown in a range of prior studies [5–7, 19] to have significant power in explaining geographic patterns of poverty and/or malnutrition, and consider in particular location/remoteness, meteorological, vegetative, market food price, and conflict data.

### 2.1 DHS malnutrition and asset poverty data

Our key poverty and malnutrition outcome indicators come from Advancing Research on Nutrition and Agriculture (ARENA) [23] aggregated DHS data or directly from the DHS [24]. ARENA is a Bill and Melinda Gates Foundation funded effort to “close important knowledge gaps on the links between nutrition and agriculture”. To that end, the ARENA project has created, and made freely available to the public, a database that combines DHS nutrition data with georeferenced agricultural and geographic data matched to the DHS data, which have modest randomized offsets to ensure respondent anonymity. The DHS offers repeated, internally comparable, nationally representative cross-sectional data on the health, welfare, and nutrition of households and individuals across 90 countries. The sample size, country, and dates of the DHS data we extracted from both ARENA and DHS are detailed in S6 Table in S1 File.

We extract from DHS and the ARENA aggregates cluster or enumeration area (EA) level estimates of poverty and malnutrition prevalence. Each EA corresponds to a roughly 10 km squared region, whose location is reported as the centroid of the EA plus a random 10 km offset designed to protect anonymity. Each of our outcomes are weighted by household survey sampling weights (with the exception of the data on women’s underweight BMI, which was weighted with individual survey sampling weights), and we consider in particular the following five outcomes:

1. Asset poverty: households in the poorest quintile of the asset-based comparative wealth index, defined as an asset index score ≤ -0.9080 (FTF 2018);
2. Child stunting: children under five years of age whose height-for-age z-score is < -2.0 standard deviations below the median on the World Health Organization (WHO) Child Growth Standards;
3. Child wasting: children under five years of age whose weight-for-height is < -2.0 standard deviations below the median on the WHO Child Growth Standards;
4. Healthy weight: children under five years of age whose weight-for-height falls in the interval [-2.0,2.0] standard deviations from the median on the WHO Child Growth Standards;
5. Underweight women: those ages 15 to 49 whose body mass index (BMI) < 18.5.

These five indicators serve as our dependent variables. Asset poverty prevalence estimates were calculated by comparing country-year specific DHS asset indices to the 2018 Feed the Future comparative wealth index threshold of -0.9080. This approach means that in same cases poverty prevalence will be overestimated and in other cases underestimated relative to a globally standardized, time invariant index. The next three indicators were compiled from raw DHS data by ARENA, and spot checked by us through comparison to DHS summary statistics provided in each DHS survey’s country report, while we estimated the women’s underweight prevalence series directly from DHS surveys.

In the following subsections, we detail a suite of input features (covariates) used for prediction of these prevalence estimates. We also extract from DHS the physical location (latitude and longitude) of each cluster EA, which allow for georeferencing of other inputs to enumeration locales, noting that EA latitudes and longitudes have been randomly offset by DHS by as many as 10km to protect the identities of survey respondents. We also include the date of a given survey as a feature, capturing the possibility of year-specific shocks common to all DHS clusters in a given survey round, and include an indicator variable on whether the cluster is urban or rural.

While DHS data are not designed to be representative at the cluster (EA) level, we use the EA as our unit of analysis because it is the smallest unit of observation in the survey for which geographic data (latitude and longitude) are available. The DHS cluster sampling strategy should also yield near-representative prevalence estimates at EA level. A consequence of working with EA level data drawn from a survey which is not intended to be representative at this level of aggregation is increased sampling noise, however, which makes the task of poverty and malnutrition prevalence prediction more challenging. Working with EA level data nevertheless has the clear advantage of enabling poverty and malnutrition monitoring at finer spatial scales, and providing larger sample size for model training, than national or larger, subnational adminitrative unit analysis would allow. Finally, we also note that DHS EAs are not fixed across surveys, meaning that the exact clusters surveyed by DHS may change from year to year, further challenging prediction, and making the usage of direct autoregression infeasible.

### 2.2 Physical geography covariates

Our first set of covariates describe the physical geography of locations, and are intended to differentiate typically wealthy urban EAs from those in more agrarian or rural locations. In particular, we extracted the following features from the ARENA data series compilation:

- Travel time to the nearest city with a population of 500,000 or more persons, as derived from IFPRI’s modeled estimates of market accessibility surface globally as the travel time from household locations to the nearest city, as described in [25–27].
- Percent tree cover data produced by [28] and presented at 1 km spatial resolution;
- Pasture coverage data present at 5 minute (∼10 km) spatial resolution drawn from Ramankutty et al’s [29] data series on agricultural lands in 2000;
- Altitude measured as the pixel elevation in meters above/below sea level; the data are drawn from Shuttle Radar Topography Mission data made available by the NASA Jet Propulsion Laboratory and the California Institute of Technology
- Slope, calculated by the IFPRI ARENA team as the degree gradient of steepness.

As the spatial sampling resolution of this data varies across features and because the true EA cluster centroid has been randomly offset to protect the privacy of survey respondents, all ARENA features were resampled to a common 5 minute spatial grid by the ARENA team in preprocessing. These features are then associated with DHS EAs by matching to each EA the feature values that are physically closest to the EA’s centroid in space, and are assumed constant for the duration of our analysis.

### 2.3 Food price data

As demonstrated by global food price spikes during the 2007–12 period vividly demonstrated, food prices can affect poverty and malnutrition patterns over space and time, as high food prices hurt poor urban residents and rural net food consumers who tend to be smallholders or landless [30]. Open access food price data may therefore be useful for monitoring changes in poverty and malnutrition patterns in a timely manner.

We collect food price data from the Food and Agriculture Organization (FAO) Food Price Monitoring and Analysis site [31], which provides monthly market-level data for major food commodities in most countries. S7 Table in S1 File summarizes these data by the number of food types monitored, number of geographic markets included, whether the data reflect retail or wholesale prices, and the first available observations in the time series, for each country. We also display in S2 Appendix in S1 File maps showing the geographic locations of monitored markets alongside DHS enumeration areas. Noting that both the value and volatility of food prices can impact poverty and malnutrition prevalence, we associate with each DHS enumeration area as features both the mean and variance of each food commodity price recorded by FAO within that survey’s country, measured in USD per kg (with the exception of Bangladesh, where prices are reported per ton). Food price means and variances are computed over a one-year window prior to the beginning of each DHS survey, with market locations georeferenced to the centroid of that market’s resident city, and with incomplete records excluded from analysis. Food price features are then assigned to EAs in a spatially ordered fashion, with the n-th set of food price features coinciding with those food price features extracted from the n-th nearest market to the EA.

### 2.4 Solar-induced chlorophyll fluorescence data

The quality and abundance of locally produced crops can have a direct impact on the poverty and malnutrition status of households. Solar-induced chlorophyll fluorescence (SIF), an optical signal emanating from the core of plants’ photosynthetic machinery, directly encodes information about crop photosynthesis [32]. Thanks to its mechanistic linkage to crop photosynthesis, SIF has the potential to be scalable for yield estimation across crop types [33, 34] in contrast to conventional greenness indices such as the enhanced vegetation index (EVI) or normalized difference vegetation index (NDVI) which can require crop specific calibration to adequately capture vegetation growth. Further, SIF is less sensitive to atmospheric contamination as it is retrieved from the in-filling of narrow Fraunhofer absorption features, than conventional greenness measures estimated from broadband reflectances [35–37]. These characteristics makes SIF an ideal measure of crop yield and health, as evidenced by its efficacy in yield prediction for both US Corn Belt and Australian wheat production [38, 39].

The recent advent of satellite based RS methods for SIF measurement [40–42] allows us to easily incorporate vegetative health into our model. We specifically use a new, long-term, high-resolution, SIF time series developed by Wen et al. [43]. This time series is recorded at monthly, 0.05 degree resolution, and uses data fusion techniques to both downscale and merge SIF retrievals from the Scanning Imaging Absorption Spectrometer for Atmospheric Chartography (SCIAMACHY) and the Global Ozone Monitoring Experiment-2 (GOME-2) [44] to construct a long-term, high resolution, precise dataset, from 2003 to 2018, which has been independently validated with both ground and airborne measurements. Its theory-based appeal motivated the use of SIF in our predictive pipeline. Sensitivity analysis found minimal differences in predictive performance when using SIF rather than more commomnly-used, but atheoretical NDVI measures.

Given the pronounced seasonality typical to agricultural production, alongside populations’ adaptations to these seasonal patterns, we assume that deviations from seasonally typical (mean) SIF conditions will serve as a better indicator of imminent food and poverty crises than will raw values. From raw SIF readings, we thus construct a set of four z-scores. These four z-scores each correspond to one of of four non-overlapping, three month, periods, roughly approximating seasons. Z-scores for a given location and season are computed using the sample mean and sample standard deviation of all raw SIF values observed during that season, and which are within 100 km of the target location. For each EA, we then assign as feature the mean of the four seasonal z-scores generated from the four seasons immediately prior to survey start date, and whose sensing location is closest to the enumeration area in space.

### 2.5 Land surface temperature data

Land surface temperatures (LST) are often estimated from satellites for purposes of drought and vegetation stress monitoring in agricultural systems [45]. Spatiotemporal variation in LST can reflect the variation in the physical processes of land-atmosphere interactions, which can in turn affect both plant evapotranspiration and surface moisture [46]. Historical satellite derived LST products have relatively coarse spatial resolutions, e.g., 0.05 degrees or worse, and suffer from spatiotemporal bias and inconsistencies when assessed at monthly scale due to the appearance of clouds and infrequent observation [47, 48].

We therefore use the new, longer-term, and higher-resolution, LST series, MYD11A1 (daytime), constructed from the Afternoon Satellite Aqua overpass. This series contains monthly composites of daily LST observations, at 1km resolution, between 2003–18 [49]. To ensure sufficient coverage, satellite pathing is controlled to ensure observation times are close to the time of daily maximum LST, while missing or anomalous readings caused by clouding are imputed using a physics-based, diurnal, temperature cycle model, which is evidently effective [50–52].

As recent studies suggest that maximum temperature is a more useful predictor of surface droughts, and thus shocks to poverty and malnutrition prevalence, than mean temperature at both annual and monthly scales [49, 53], we extract from this series in particular monthly composites of daily maximum LST for use in modeling. Again assuming that deviations in maximum monthly LST will be more indicative of poverty and malnutrition status due to population adaptation to typical temperatures, we again apply the same seasonal normalization procedure used for SIF data assignment, and again assign to each EA the mean of the four maximum LST z-scores generated during the four seasons immediately prior to survey start date, and which are sensed closest to the EA in space.

### 2.6 Precipitation data

As precipitation and rainfall are intimately linked to both crop production and water availability, we use monthly, 0.05 degree, precipitation data from the Climate Hazards group Infrared Precipitation with Stations (CHIRPS) as a metric for water availability [54]. CHIRPS data is produced based on satellite and station observations, together with precipitation climatology informed imputation, as described in [55]. These data have been widely validated and employed to characterize water availability and detect drought, especially on the African continent [55–60]. We again use as features seasonally normalized readings, and again assign to each EA the mean of the four CHIRPS derived z-scores generated during the four seasons immediately prior to the survey start date that are sensed closest to the EA in space.

### 2.7 Conflict data

Armed conflict and political instability are well known to cause adverse shocks in food security and poverty status [61]. To incorporate these phenomena, we source data from the Uppsala Conflict Data Program (UCDP) [62, 63] which provides descriptions of violent events, or incidents wherein “armed force was used by an organized actor against another organized actor, or against civilians, resulting in at least 1 direct death at a specific location and a specific date”. To adhere to this definition, UCDP data include violent events only if there are clear estimates of fatalities and a clear indication of actors involved.

In preprocessing, we remove events that lack a georeference (roughly 10 percent of events), or timestamp (roughly 3 percent). Because violent conflict commonly represents country-level political instability and because the conflict data are relatively sparse, we treat the conflict data in a country-specific but otherwise spatially agnostic framework, and associate with each given survey in a particular country the number of violent events and number of resulting casualties occurring in the year prior to the DHS survey start date. As many violent events are part of a more protracted conflict, these counts also include all longer (greater than 1 day) events whose terminal date lies within the one-year window prior to the start date of each DHS survey.

### 2.8 Additional preprocessing

As a final stage of preprocessing, we remove any features that are missing for more than 20 percent of a given test/train regime (soon to be discussed), and remove datums that contain any additional missing features or outcomes. Due to occasional variation in the exact commodity prices reported by FAO within each country across survey years, this former decision causes the occasional omission of market price data when analyzing certain DHS surveys, while the latter results in omission of the 2012 and 2014 DHS surveys in Senegal, where underweight female BMI data are missing, alongside a small reduction in sample size.

## 3 Methodology

### 3.1 Modeling pipeline

To assess whether competitive predictions for poverty and malnutrition prevalence can be produced without the need for deep learning methods, we consider a random forest (RF) model. Random forests are among the most commonplace ML algorithms, and can be easily implemented using widely available off-the-shelf packages in many programming languages. We explore both independent RFs, which will predict each poverty or malnutrition outcome separately, as well as multivariate RFs, which employ joint estimation of outcomes.

As interventions targeting poverty and malnutrition prevalence are typically performed at country and year specific levels, and due to variation in both the number and meaning of food price features across countries, we assume a survey (i.e. country and year) specific relationship between model inputs and prevalence rates. For countries *c* = 1, 2, …, 11, we denote by $\left({\text{x}}_{i}^{t,c},{\text{y}}_{i}^{t,c}\right)\in \left(R^{p^{c}},R^{5}\right)$ respectively our *p*^*c*^ input features specific to country *c* and country agnostic outcomes (prevalences), with *i* = 1, …*n*^*t*,*c*^ counting the total number of EAs surveyed by DHS in country *c* during year *t*. Our survey specific model for joint prediction of the five target prevalence rates can then be written in a signal-plus-noise formulation:

$$\begin{array}{c} {\text{y}}_{i}^{t,c}={\text{f}}^{t,c}\left({\text{x}}_{i}^{t,c}\right)+\epsilon_{i}^{t,c}, \end{array}$$ (1)

where $\epsilon_{i}^{t,c}$ are assumed i.i.d. across observations *i* for all years and countries, *t* and *c*, respectively, with mean **0**, covariance Σ^*t*,*c*^.

To allow for incorporation of possible dependencies between poverty and malnutrition prevalences, we model each mapping **f**^*t*,*c*^ via a (multivariate) Mahalanobis random forest (MRF) [64–66], which extends the traditional univariate random forest model to a multivariate setting in which joint prediction of outcomes is performed through the explicit inclusion of outcome dependencies that are estimated in training. Prediction within the context of an MRF is a straightforward extension of the traditional setting, with terminal nodes of a given tree containing as prediction the componentwise mean of resident outcome vectors, while forest predictions at a testing point **x**^*t*,*c*^ are analogously the componentwise average over the individual predictions $h_{k}^{t,c}\left({\text{x}}^{t,c}\right)$ of each tree within the forest ensemble:

$$\begin{array}{c} \begin{array}{c} {\hat{\text{f}}}^{t,c}\left({\text{x}}^{t,c}\right)=\frac{1}{T}\sum_{k=1}^{T}h_{k}^{t,c}\left({\text{x}}^{t,c}\right),\\ h_{k}^{t,c}\left({\text{x}}^{t,c}\right)=\frac{1}{|L_{k}\left({\text{x}}^{t,c}\right)|}\sum_{i=1}^{n^{t,c}}{\text{x}}_{i}^{t,c}\cdot I_{L_{k}\left({\text{x}}^{t,c}\right)}\left({\text{x}}_{i}^{t,c}\right), \end{array} \end{array}$$ (2)

where *L*_*k*_(**x**^*t*,*c*^) denotes the leaf containing **x**^*t*,*c*^ in tree *k* = 1: *T*, |⋅| denotes cardinality, and where *I*_*A*_(⋅) is an indicator function on the set *A*, so $I_{L_{k}\left({\text{x}}^{t,c}\right)}\left({\text{x}}_{i}^{t,c}\right)=1$ if the *i*th input observation is in the same leaf as the testing point, and 0 otherwise.

The primary distinction of the MRF then comes in training, with node splits now chosen via Mahalanobis distance:

$$\begin{array}{c} C_{L}\left(X^{t,c},{\text{Y}}^{t,c}\right)=\sum_{i=1}^{n^{t,c}}{\left({\text{y}}_{i}^{t,c}-{\bar{\text{y}}}_{L}\right)}^{\prime }\Lambda^{t,c}\left({\text{y}}_{i}^{t,c}-{\bar{\text{y}}}_{L}\right)\cdot I_{L}\left({\text{x}}_{i}^{t,c}\right), \end{array}$$ (3)

where ${\bar{\text{y}}}_{L}$ denotes the mean response vector within leaf *L*, and with Λ^*t*,*c*^ denoting the precision matrix, i.e., the inverse of the covariance matrix Σ^*t*,*c*^. The modified cost function (3) can then be reinterpreted as replacing the traditional variance criterion of random forest regressor with a sum of variances across independent output dimensions, following their decorrelation as specified by Λ^*t*,*c*^. This allows for splits to be chosen in a way that jointly minimizes variance across outcomes while incorporating output dependency, ideally improving predictive performance by leveraging outcome dependencies.

### 3.2 Hyperparameter selection

We set *T* = 2,000 as our forest size, then chose random forest hyperparameters of max tree depth (*d*) and feature downsampling rate (*dsr*) via five-fold cross validation on training data. While this cross validation was initially performed separately for each survey, and separately for both sequential and contemporaneous frameworks, which will be discussed in the next section, we ultimately chose to select a single set of shared hyperparameters $d=4,dsr=\frac{1}{3}$, corresponding to the modally selected hyperparameter values across all surveys and predictive frameworks, causing a negligible change in performance at the benefit of reproducibility and parsimony.

Since the error covariance matrices Σ^*t*,*c*^ are in general unknown, we estimate them by first fitting a collection of univariate random forests for each country, year, and outcome, independently. Training residuals from these models were then used to directly estimate Σ^*t*,*c*^, alongside its inverse Λ^*t*,*c*^, for use in Eq 3, in an approach analogous to Feasible Generalized Least Squares. In testing, results from these independent random forest models are then used to establish a baseline against which to compare our joint model.

## 4 Results and analysis

We now consider two distinct predictive tasks. The first, which we term sequential nowcasting, is intended for use in early warning systems, and considers the sequential generation of near future forecasts of future poverty and malnutrition prevalence using historical outcome data and current (i.e. present) inputs. The second, which we term contemporaneous prediction, is intended to inform geographic targeting in poverty or malnutrition interventions, or to be used for monitoring and evaluation purposes, and is used when one observes outcomes in only a sample of locations, which are to be generalized to a larger spatial domain based on these current survey year observations. Although these two tasks can use similar data and methods, we emphasize that these distinct use cases are not necessarily interchangeable, and we find considerable differences in predictive performance across these two regimes, underscoring the importance for agencies and analysts to define their intended task in modeling and evaluation.

### 4.1 Sequential nowcasting

To assess the ability of our model to generate near future forecasts of poverty and malnutrition prevalence for use in early warning systems, we consider a sequential prediction framework in which, for each country surveyed in year *t* we predict poverty and malnutrition prevalence during year *t*, which acts as our testing set, using only historical training data drawn from previous DHS surveys in years *t*′ < *t* occurring in the same country, alongside RS input features from year *t*. While minimally studied in the existing literature, we find that RFs, used in conjunction with open access data, can produce relatively accurate forecasts of certain prevalence rates, with modest improvements in forecast accuracy gained via our joint estimation approach, for certain prevalences.

We evaluate model performance for each indicator via out-of-sample *r*^2^ and root mean squared error normalized (NRMSE) by (in-sample) observed prevalence range:

$$\begin{array}{c} r_{j}^{2}=1-\frac{\sum_{i=1}^{n}{\left(y_{i,j}-{\hat{y}}_{i,j}\right)}^{2}}{\sum_{i=1}^{n}{\left(y_{i,j}-{\bar{y}}_{i,j}\right)}^{2}}, \end{array}$$ (4)

$$\begin{array}{c} {\text{NRMSE}}_{j}=\frac{\sqrt{\frac{1}{n}\sum_{i=1}^{n}{\left(y_{i,j}-{\hat{y}}_{i,j}\right)}^{2}}}{\max_{i=1:n}\left(y_{i,j}\right)-\min_{i=1:n}\left(y_{i,j}\right)}, \end{array}$$ (5)

where *j* denotes the *j*th prevalence *j* = 1: 5 and where *i* denotes the *i*th EA area considered in a given test-train framework.

**Fig 1:**
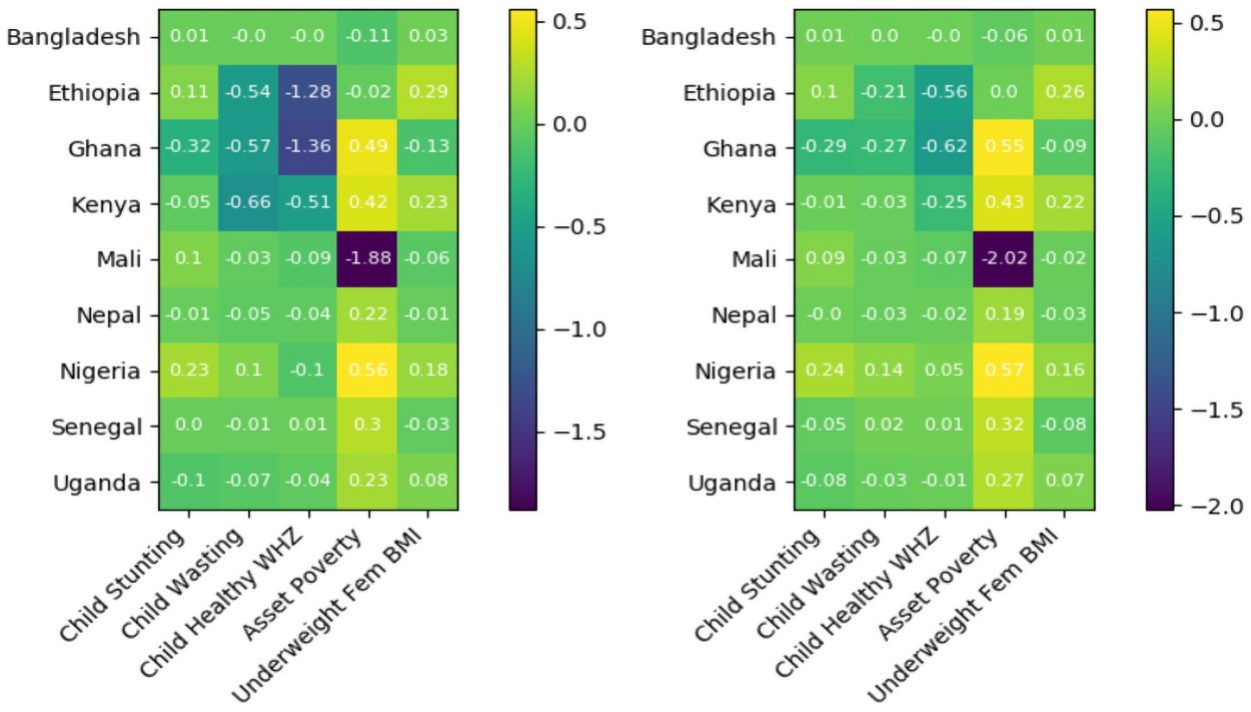
*r*^2^ for sequential poverty and malnutrition prevalence nowcasting indexed by country. (Left) Independent RF (Right) MRF. Scores are computed by pooling predictions across all surveys within each country, with shared testing and training folds used across all models.

Predictive performance is assessed at three levels of aggregation. At the coarsest scale, we assess fully aggregate results, which are computed by pooling all predictions across all surveys, displayed in Table 1. We next assess predictive performance at the individual country level, wherein predictions are pooled across all surveys within each country, displayed in Fig 1, and with Table 2 reporting mean country level performance weighted by relative country survey size. Finally, at the finest level of aggregation, we report individual survey level performance, with results displayed at length in S1 Appendix in S1 File.

**Table 1:** Aggregate out-of-sample *r*^2^ and NRMSE for sequential nowcasting, indexed by methodology and prevalence.

		Child Stunting	Child Wasting	Healthy Weight	Asset Poverty	Underwt Women
IRF	*r* ^2^	0.07	-0.01	-0.21	0.21	0.31
MRF	*r* ^2^	0.08	0.10	-0.04	0.21	0.29
IRF	NRMSE	0.21	0.15	0.16	0.26	0.17
MRF	NRMSE	0.21	0.12	0.15	0.27	0.12

Scores are computed by pooling all predictions across all surveys, with shared testing and training folds used across all models.

**Table 2:** Mean country level out-of-sample *r*^2^ and NRMSE for sequential nowcasting, indexed by methodology and prevalence.

		Child Stunting	Child Wasting	Healthy Weight	Asset Poverty	Underwt Women
IRF	*r* ^2^	0.01	-0.24	-0.39	0.12	0.11
MRF	*r* ^2^	0.02	-0.04	-0.17	0.13	0.10
IRF	NRMSE	0.21	0.16	0.17	0.24	0.17
MRF	NRMSE	0.21	0.14	0.16	0.24	0.17

Scores are computed by taking a size weighted average of individual country level performance, with shared testing and training folds used across all models.

As will be later shown, though nowcasting of poverty and malnutrition prevalence appears to be a considerably more challenging task than contemporaneous prediction, we note decent performance in prediction of asset poverty and underweight women prevalence, in comparison to our modest predictions for child stunting and wasting, while our least skilled predictions are of healthy weight children. Because healthy child weight is the complement of both child wasting and obesity, and because our predictions for child wasting are superior to our predictions for child healthy weight, this finding indicates that child obesity may be especially hard to predict with these data and methods. This hypothesis seems in line with theoretical foundations, as obesity appears strongly related to health insults in utero, lifestyle preferences, and cultural environment, none of which are readily captured by any of the data we use [67, 68]. Alternatively, it is interesting to note in S26 Fig in S1 File that the average percent change across surveys in DHS prevalence rates is relatively low for malnutrition indicators. Thus, an alternative explanation for our higher performance in prediction of poverty relative to malnutrition prevalence is that because there is little inter-survey variation in malnutrition prevalence, the sample means of malnutrition prevalences from prior survey will be reliable predictors of future prevalence, and so outperforming this baseline (i.e., attaining a high *r*^2^) will be more challenging. Further support for this narrative is given by our relatively low NRMSEs across all prevalences, despite weaker *r*^2^s. This interpretation is consistent with the hypothesis that most observed malnutrition in these settings is chronic rather than transitory.

Joint estimation of outcomes seems to produce modest improvements in nowcasting of child nutritional outcomes, relative to independent RF modeling, as measured by *r*^2^, due to the clear correlation between these indicators. We also note that for both independent random forest and MRF models, our relatively low NRMSEs indicate that these models can produce forecasts capable of reasonably informing aid planning and policy, being accurate to within roughly 10 to 25 percent of the observed range of each indicator. We interpret these results as showing promise for the use of alternative ML methods and open access data for early warning, and motivating joint estimation as a potential direction of future work.

As evident in Tables 1 and 2, and in Fig 1, we observe a considerable drop in performance, as measured by *r*^2^, when assessing our results at more granular scales. This effect becomes more pronounced at the even finer survey level assessment scale, detailed in S1 Appendix in S1 File, where our model exhibits high variance in predictive performance on individual surveys, with a significant deterioration in mean performance. Considering these findings in the context of individual survey sizes listed in S6 Table in S1 File, we believe this drop in predictive performance at finer assessment scales arises largely from extremely poor performance on a subset of three outlier surveys, each of which is the first sequential testing year in countries with relatively small sample size DHS surveys. This hypothesis is further supported by directly comparing the impact of survey size on survey level predictive performance, displayed in S6 Fig in S1 File, where we observed a clear positive association between survey size and predictive performance, and by noting our survey size weighted, mean, country-level performance remains well above the lower tail of individual country-level performance.

In the context of our task, it should come as little surprise that attempting to generate nowcasts from a small and several years old training set will generate inaccurate predictions. This shortcoming should therefore diminish steadily as the history and/or sample size of DHS surveys grow, and implies that the reliability of our nowcasts depend heavily on the quality of the underlying survey data upon which they are built. While this finding indicates a shortcoming of our method relative to deep or transfer learning approaches which are less reliant on sample size, it also motivates the need for further survey efforts and feature collection to facilitate and enhance the prediction of poverty and malnutrition prevalence via statistical methods and RS data.

An alternative explanation for these findings is that failure to incorporate spatial autocorrelation in our outcome variables might degrade predictive performance when moving from large scale analysis to finer spatial domains, where spatial associations exert stronger influence on outcome variability, resulting in worse relative fit [69]. Allowing for the possibility of spatial autocorrelation of outcomes therefore seems a fruitful direction for future work. Despite these findings, we note qualitatively similar NRMSE at both aggregate, country, and survey level scales (see S1, S2 Tables in S1 File). This suggests that our model can generate reasonably accurate predictions of key malnutrition and poverty indicators at different scales of analysis, even with relatively sparse data.

Despite our method’s shortcomings, our results seem broadly comparable to prior related works. While competing methods for sequential forecasting or nowcasting of poverty and malnutrition prevalence are sparse, the most directly comparable work may be Yeh *et al*. [13], who predict changes in DHS asset poverty over time for a set of 23 sub-Saharan African countries using multispectral, convolutional neural networks. These authors report cluster level out-of-sample *r*^2^ = 0.18, with minimal difference when predictions are assessed in aggregate or averaged over individual countries, as compared to our aggregate and country-level mean *r*^2^s of 0.21 and 0.14, respectively. We emphasize however that direct comparison of results is impossible due to differences in both the encoding of poverty status, scope of analysis, and the important distinction between nowcasting and prediction of change in prevalence.

Nevertheless, this comparison seems to establish a basic credence to our results, indicating that RF and MRF models can produce results competitive with those generated by deep or transfer learning based methods, when applied to the right data, even with infrequent observations. Given the relative ease of implementation of our MRF approach and the open access to our data, this seems a comparatively easy-to-implement, tolerably accurate, first pass method that agencies might use as part of a broader early warning system. Such preliminary findings could perhaps be usefully supplemented with proprietary data and transfer learning based approaches. We also note that although we had to exclude central American countries Guatemala and Honduras from our sequential prediction because they each had only a single year of georeferenced DHS data available, our work extends the sequential prediction of poverty and malnutrition prevalence beyond sub-Saharan Africa, to Bangladesh and Nepal, demonstrating that simple models of these phenomenon can be easily developed and applied globally with modest accuracy which could be further improved in future works.

**Fig 2:**
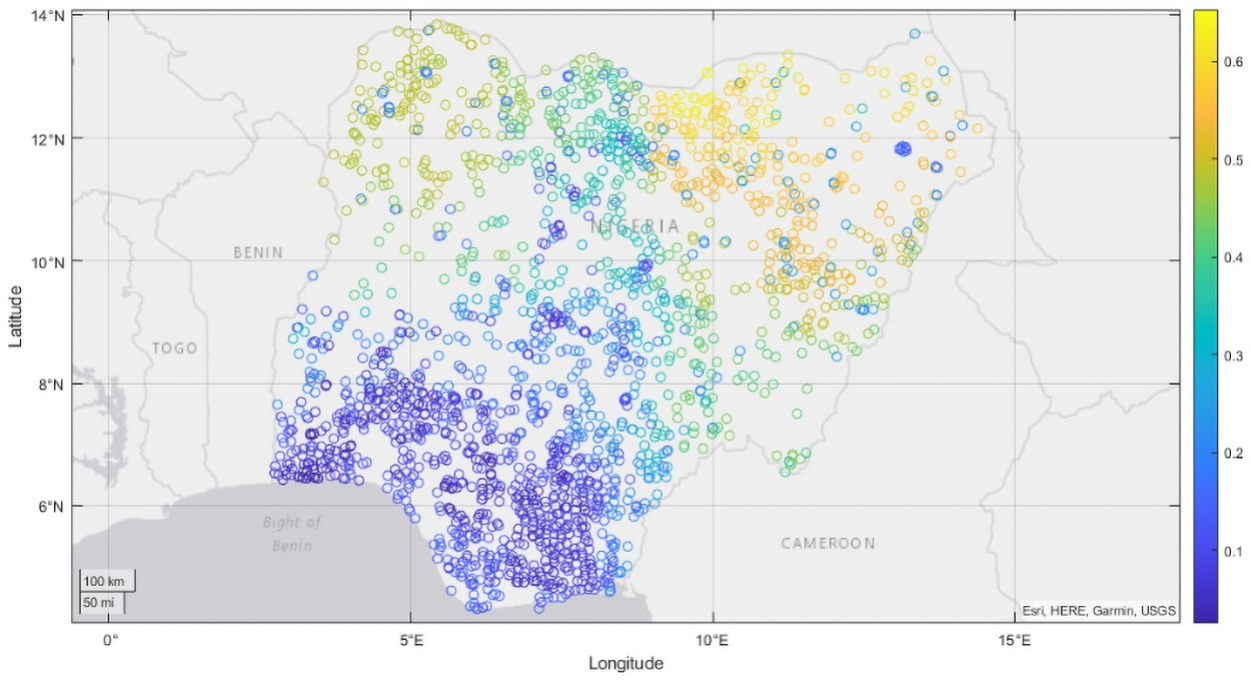
Mean nowcasts for asset poverty prevalence for Nigeria, 2013. Within survey *r*^2^ is 0.56.

**Fig 3:**
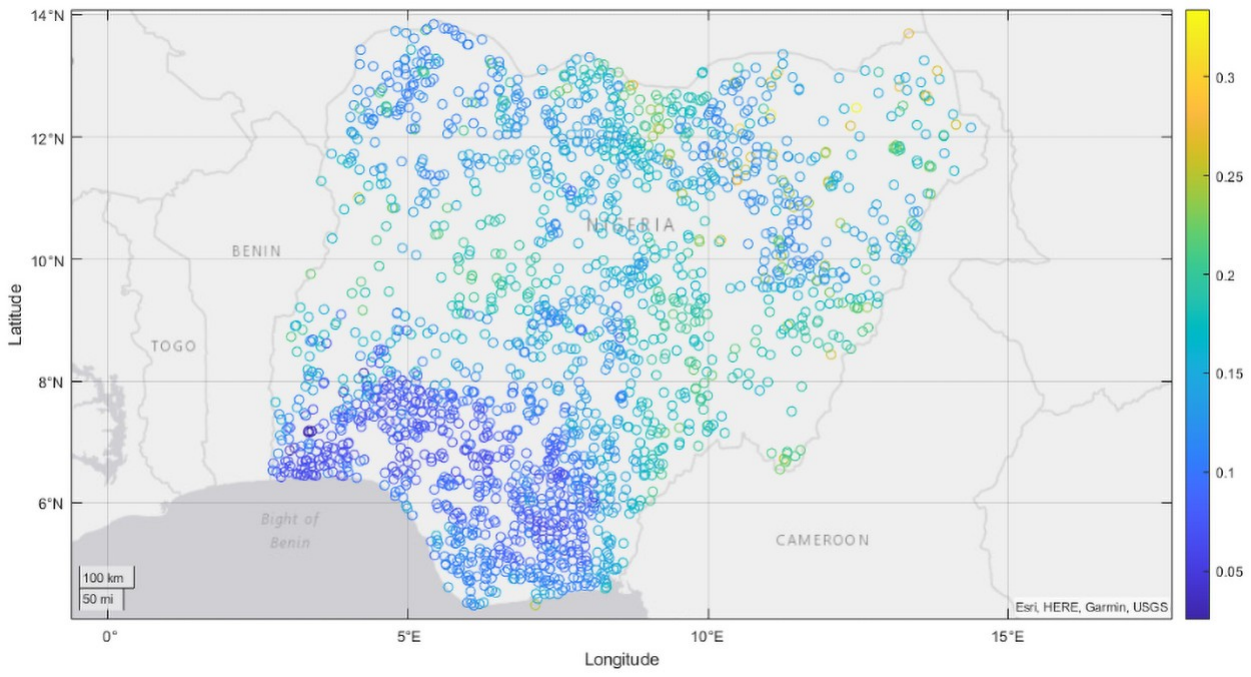
Uncertainty in asset poverty prevalence for Nigeria, 2013. Uncertainty is measured as the standard deviation of asset poverty prevalence prediction across each tree in our MRF.

To demonstrate how the forecasts of our MRF model could be used to provide policymakers with visual, georeferenced, predictions of poverty and malnutrition prevalence, Figs 2 and 3 provide a visual depiction of a near future, spatial, forecast for asset poverty prevalence across Nigeria in 2013, generated using 2008 DHS survey data, with additional nowcast maps displayed for each country in in S3 Appendix in S1 File. Such maps are widely used in early warning systems because they provide effective visualization tools for policymakers, and inform the spatial allocation of scarce resources by logisticians [70]. When produced repeatedly over time, these maps can also be used to provide baseline, midline and endline measures for monitoring and evaluation purposes, and allow for estimation of poverty and malnutrition status in areas where reliable survey data may be unavailable.

To indicate uncertainty or reliability in our predictions, we also report the standard deviation of our predictions across each individual tree in our MRF ensemble. In the case of Nigeria, we find our predictions to show qualitative agreement with contemporaneous products developed by the Famine Early Warning System for Nigeria in 2013 [71], where the northwestern and northeastern regions exhibited the greatest concentrations of acute malnutrition and poverty, despite our nowcasts being produced in advance of 2013 survey data. Examining Fig 3, we see that our predictions seem most reliable in areas which are dense in survey coverage and which exhibit minimal spatial variation in prevalence, with our least reliable predictions occurring in the eastern portion of Nigeria, where survey coverage is minimal, and in the north where our predictions for poverty prevalence exhibit spatial heterogeneity, indicating that such regions could be a focus of future survey efforts.

### 4.2 Contemporaneous prediction

A different, and typically easier, task is to use survey observations to predict contemporaneous values for unsurveyed locations. As discussed earlier, the resulting contemporaneous predictions can be very useful for monitoring and evaluation or geographic targeting purposes.

We next apply our MRF approach to a cumulative, contemporaneous, predictive task, wherein, for each country surveyed in year *t*, all data from previous survey rounds *t*′ < *t*, along with 80 percent of the data from survey year *t*, are used for model training, with the remaining 20 percent of year *t* data held out for testing. Testing and training is performed five times for each survey, corresponding to five-fold cross validation across data from survey year *t*, with all results reported corresponding to the average score computed across all folds.

**Fig 4:**
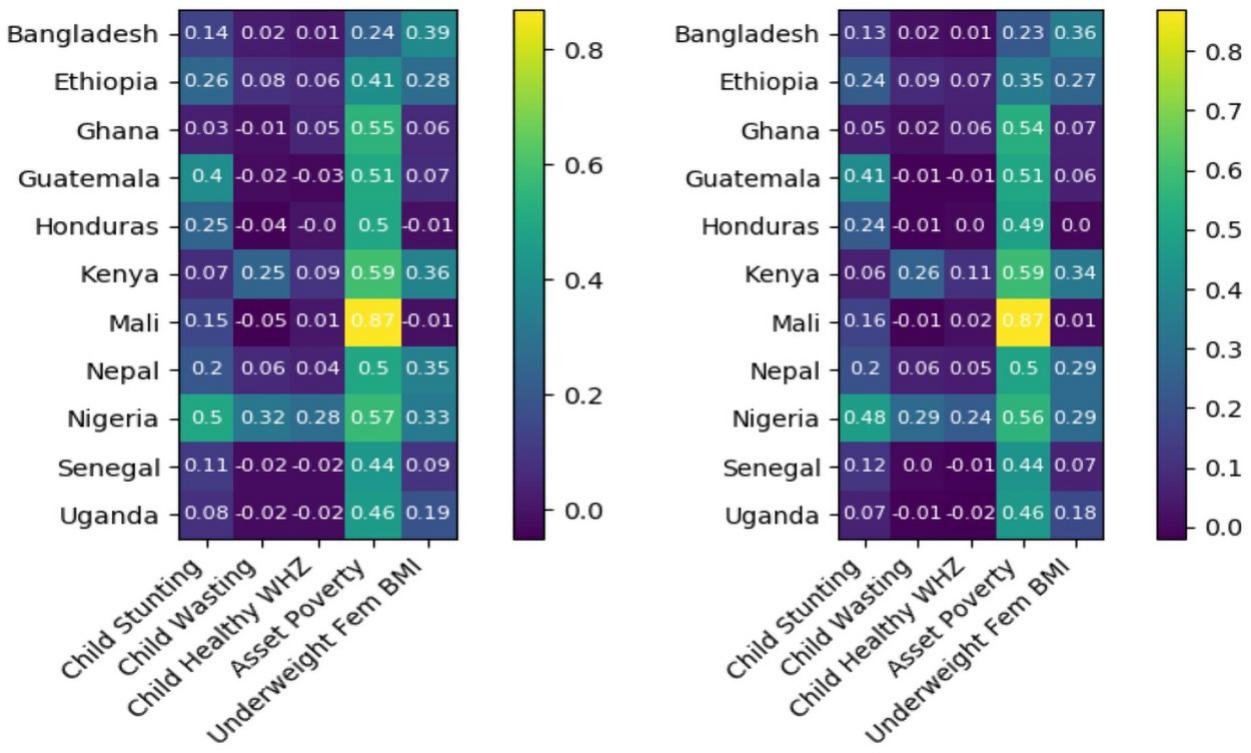
*r*^2^ for contemporaneous poverty and malnutrition prevalence prediction by country. (Left) Independent RF (Right) MRF.

In Tables 3 and 4 we again assess our predictive results for contemporaneous prediction of poverty and malnutrition prevalence via our of sample *r*^2^ and NRMSE. Again, predictions are assessed at three levels of granularity, with fully aggregate results displayed in Table 3, country-level results displayed in Fig 4 and Table 4, and with survey level results relegated to S1 Appendix in S1 File.

**Table 3:** Aggregate mean and standard deviation of out-of-sample *r*^2^ and NRMSE for contemporaneous prediction, indexed by methodology and indicator.

		Child Stunting	Child Wasting	Healthy Weight	Asset Poverty	Underwt Women
IRF	mean *r*^2^	0.28	0.23	0.17	0.58	0.48
MRF	mean *r*^2^	0.27	0.23	0.17	0.58	0.46
IRF NRMSE	mean	0.19	0.11	0.14	0.19	0.11
MRF NRMSE	mean	0.19	0.11	0.14	0.19	0.11
IRF	std *r*^2^	0.00	0.01	0.02	0.01	0.01
MRF	std *r*^2^	0.00	0.00	0.00	0.00	0.01
IRF NRMSE	std	0.19	0.11	0.14	0.19	0.11
MRF NRMSE	std	0.00	0.00	0.00	0.00	0.01

Scores are computed using predictions across all countries and survey years, with mean and standard deviations calculated across 5 folds. Testing and training folds are shared across models.

**Table 4:** Mean and standard deviations of country level *r*^2^ and NRMSE for contemporaneous prediction, indexed by methodology and indicator.

		Child Stunting	Child Wasting	Healthy Weight	Asset Poverty	Underwt Women
IRF	mean *r*^2^	0.21	0.08	0.06	0.49	0.24
MRF	mean *r*^2^	0.20	0.09	0.06	0.48	0.22
IRF NRMSE	mean	0.21	0.19	0.21	0.19	0.19
MRF NRMSE	mean	0.21	0.19	0.20	0.19	0.19
IRF	std *r*^2^	0.14	0.12	0.09	0.15	0.15
MRF	std *r*^2^	0.14	0.11	0.08	0.16	0.13
IRF NRMSE	std	0.03	0.03	0.03	0.03	0.04
MRF NRMSE	std	0.03	0.03	0.03	0.03	0.03

Scores are computed using predictions for all surveys within each country, with mean and standard deviation of performance measured across folds and countries. Testing and training folds are shared across models.

Since contemporaneous prediction makes use of all the same features as the sequential nowcasting task, while having more current data, it should, and does, prove to be a more tractable problem. We find significant improvements in mean *r*^2^ scores and more modest improvements in mean NRMSE relative to our sequential nowcasting framework, for both independent and joint random forest models, and at all levels of aggregation. Unfortunately, and in contrast to the sequential nowcasting case, we find no performance improvement from joint estimation of malnutrition and poverty prevalence rates, indicating that the leverage gained through joint estimation of target outcomes may be less relevant when testing and training data are closer in time. Thankfully, we find that simple, univariate, random forest models can produce relatively accurate, and competitive, contemporaneous, predictions of both malnutrition and poverty prevalence rates. We observe little variation in aggregate model performance, implying that at this level, or results seem robust. In contrast, when examining variability in country level performance as measured by *r*^2^ across folds and countries, we see a considerably larger degree of variation in predictive performance, consistent with Fig 4. This variability stems primarily from variation in performance across countries, with the maximum variability across folds observed for any indicator and country being.04, implying that while our model remains reliable at the country level with respect to random fluctuations in training and testing data, model performance in one country is not necessarily indicative of performance in another. We also note minimal variability in NRMSE in this context, implying that predictions remain tolerably accurate across countries. We finally note again a general reduction in mean performance when assessing model fit at a country and survey specific level, which presumably arises for the same reasons as in sequential prediction, though we find the magnitude of this performance reduction to be less extreme in the contemporaneous case.

Table 5 provides a basis for qualitative comparison between our results and related contemporaneous prediction studies that use DHS poverty or malnutrition indicators. Relative to the dirth of such studies for sequential nowcasting, we are aware of at least three such papers [8, 10, 13] which apply transfer learning and convolutional neural networks to contemporaneous prediction of DHS asset poverty measures, with the latter also offering a basis for comparison against our malnutrition predictions. Jean *et al*. [8] report out-of-sample, aggregate *r*^2^ = .59, with *r*^2^ ∈ [0.55, 0.75] for individual survey performance across single DHS surveys from five African countries: Malawi, Nigeria, Rwanda, Tanzania and Uganda. Yeh *et al*. [13] report an aggregate *r*^2^ = .67, and mean country-level *r*^2^ = .70, with predictions done at the village level, using DHS surveys across 23 African countries. Head *et al*. [10] used four surveys, conducted in Honduras, Nepal, Nigeria, and Rwanda, and report survey specific *r*^2^ ∈ [0.51, 0.74] for asset wealth, *r*^2^ ∈ [0.31, 0.47] for underweight female BMI, *r*^2^ ∈ [0.03, 0.35] for child height percentile, and *r*^2^ ∈ [−.02, −.11] for child height-to-weight percentile.

**Table 5:** Summary comparison to related works for contemporaneous prediction of DHS derived malnutrition and poverty indicators.

Paper	Child Stunting	Child Wasting	Asset Poverty	Underwt Women
Jean *et al*. [8]			0.59, 0.55–0.75^†^	
Head *et al*. [10]	0.03–0.35^†^	-0.02–0.11^†^	0.51–0.74^†^	0.31–0.47^†^
Yeh *et al*. [13]			0.67, 0.70^⋆^	
This paper	0.28, 0.21^⋆^, 0.17^†^	0.23, 0.09^⋆^, 0.08^†^	0.58, 0.49^⋆^, 0.44^†^	0.48, 0.24^⋆^, 0.20^†^

Unmarked, ⋆, and † flagged *r*^2^ values represent aggregate, mean country, and mean survey level, results respectively. Note that Head *et al*. [10] reports predictive performance for child weight for height percentage and child height percentile, which are not directly comparable to our wasting or stunting metrics, and we estimate their performance on the latter from a figure.

Again, our random forest models perform comparably to these related works. Our aggregate performance in asset poverty prediction is almost to the *r*^2^ = .59 reported by Jean *et al*. [8] but is outperformed by Yeh *et al*. [13]. Our models do less well in predicting female underweight BMI than Head *et al*. [10] but as well or better in predicting child nutritional outcomes. A clear, relative weakness of our method is evident when assessing model performance at fine (i.e. survey level) scales, falling on the low end of the performance spectrum and being clearly outdone by by Yeh *et al*. [13], indicating again the relative strength of transfer learning methods which are less reliant on sample size. Again emphasizing the challenges of direct comparison due to differences in the exact encodings of DHS derived outcomes, we again find RF methods and open source data can produce competitive results for contemporaneous prediction of poverty and malnutrition in aggregate, with the caveat of underperformance at country or survey specific levels of analysis, relative to state of the art. As in sequential forecasting, we interpret these comparisons as suggesting that RF models and open source data can provide reasonable first pass estimates of poverty and malnutrition prevalances, which can supplement contemporaneous survey-based estimates for geographic targeting and for monitoring and evaluation purposes, while being potentially easier for agencies to adopt than competing methods.

### 4.3 Feature importance

**Fig 5:**
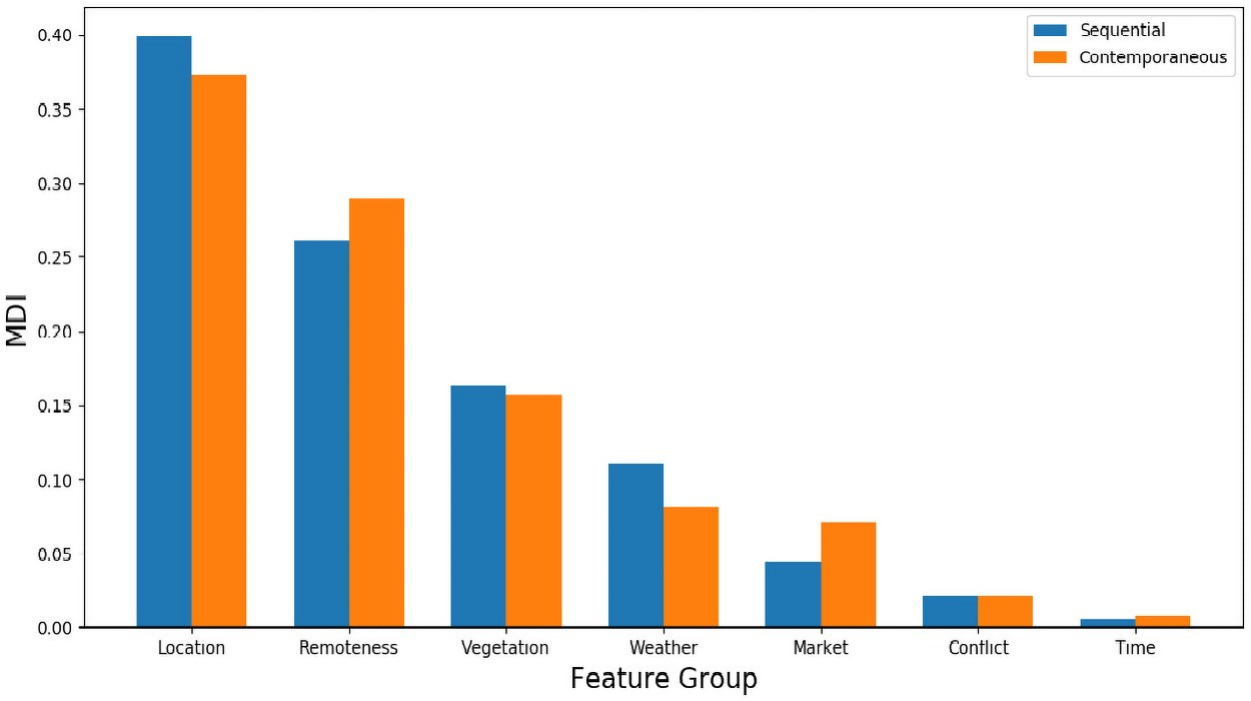
Mean MDI over all surveys, grouped by data type. Location refers to survey latitude, longitude, altitude, and slope. Remoteness indicates urban-rural status and distance to nearest major city. Vegetation includes pasture coverage, tree coverage, and SIF readings. Weather includes CHRPS and LST data. Market contains all food price data. MDI for each data type is computed by combining individual feature MDIs for each feature within that category.

**Fig 6:**
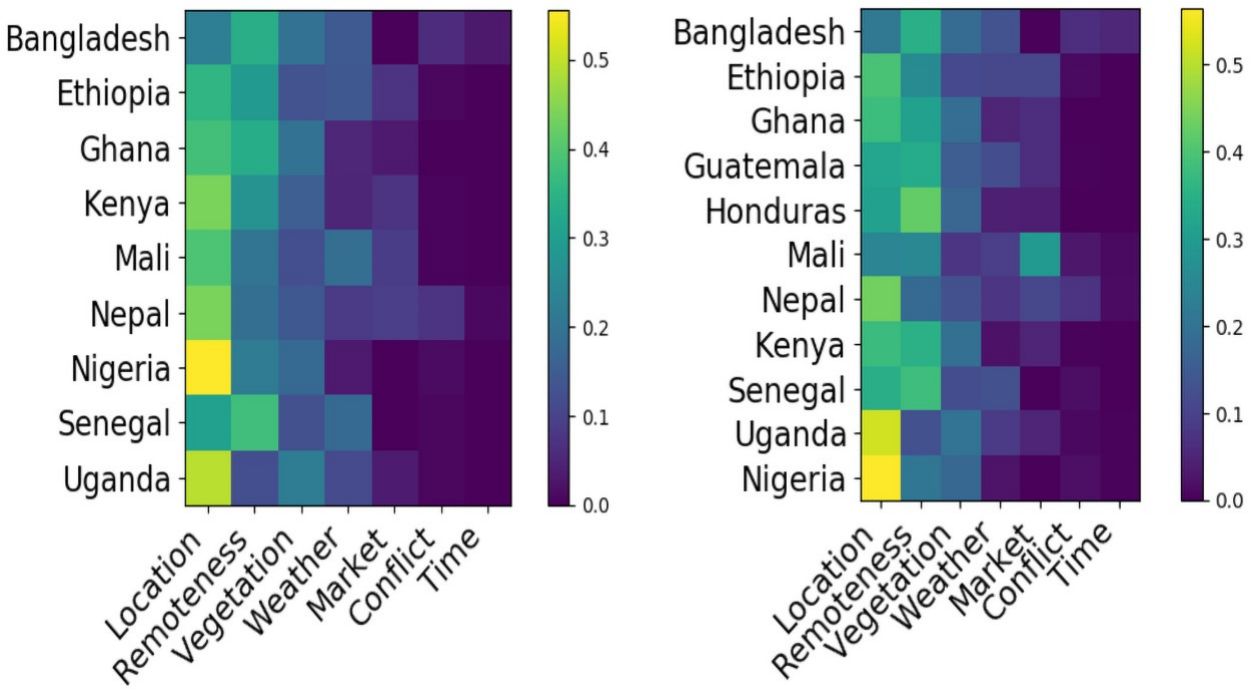
Mean relative variable importance across survey years, indexed by country and data source. (Left) Sequential (Right) Contemporaneous.

Understanding what features are most informative of poverty and malnutrition status can help inform aid programs and prioritize future data collection efforts. We provide here a brief summary of feature variable importance. Feature importances are reported as the mean decrease in impurity (MDI) for each feature, which loosely measures the extent to which inclusion of a given feature improves the fit of our MRF model. Given a feature *x*, decrease in impurity is calculated as the product of the percentage of training samples split on *x*, times the resulting decrease in model cost (variance) resulting from such splits, with MDI defined as the average of these quantities across the forest ensemble. MDI is reported separately for both our sequential and contemporaneous frameworks, with Figs 5 and 6 displaying the average MDI for each feature across all surveys (i.e. in aggregate), and across all surveys within each country, respectively.

Fig 5 shows that physical geography features indicating EA location and remoteness account for the largest share of predictive skill in both contemporaneous prediction and sequential nowcasting, indicating that the dominant action of our model is to simply assign as prediction for a given EA the mean prevalence rates observed in nearby EAs during previous survey rounds. While effective for prediction and a confirmation of the value of geographic targeting, these relationships are clearly non-causal and relatively static, and are thus somewhat dissatisfying from a policy and planning perspective. We also note however that both vegetation and weather features meaningfully contribute to skill in predicting poverty and malnutrition, particularly in the sequential nowcasting framework that is appropriate to early warning systems.

Perhaps surprisingly, given the considerable attention paid to food price shocks and conflict as drivers of poverty and malnutrition, those features exhibit comparatively little predictive power. The limited importance of food price data in particular may be partially an artifact of our data construction and cleaning process; Intra-country variation in food price data availability across surveys alongside our exclusion of features missing for more than 20 percent of EAs within a given test-train framework during preprocessing may have mechanically reduced the overall importance of these features in aggregate, implying that enhanced focus on market price data collection might still prove useful to poverty and malnutrition estimation. Alternatively, this finding may be explained by possibly poor overlap between DHS enumeration areas and market locations, which can be observed in S2 Appendix in S1 File. The limited importance of conflict data may also be explained as a result of our feature engineering, in which these features were treated in a country specific but otherwise spatially agnostic framework, limiting their discriminative ability. Alternatively, it is plausible that because conflict ridden areas may be more challenging to survey, areas in which poverty and malnutrition prevalence were driven by conflict were undersurveyed, resulting in their omission from our data, and implying that enhanced survey focus on these areas could be beneficial, albeit challenging. In contrast, the unimportance of time as a predictive feature within our random forest model is unsurprising; Given that our testing points are always drawn from a single survey year, only one branch of any tree split on time will be followed during testing, with the split itself simply acting to discard older data.

## 5 Conclusions

As they grapple with the broad consequences of the global pandemic and resulting economic disruptions, governments and development or humanitarian organizations worldwide need more than ever practical tools to inform targeting of beneficiaries, monitor and evaluate interventions, or provide early warning of prospective increases in malnutrition or poverty prevalence. While such efforts have historically been informed by surveys which can be costly and slow to produce, the recent advent of machine learning and remote sensing offers these organizations an opportunity to develop faster and cheaper methods of assessing poverty and malnutrition status. Furthermore, given limited financial and human resources, there seems widespread preference for interpretable statistical methods that can rely solely on free, open access, RS data.

In this paper, we demonstrate the viability of both contemporaneous prediction and sequential nowcasting of malnutrition and poverty prevalence using a set of features drawn from open access data sources, in conjunction with random forest methods. We found that multivariate, joint prediction of multiple malnutrition and poverty indicators modestly improved predictive performance of some prevalences in sequential nowcasting, but not in contemporaneous prediction. Given the simplicity of our joint modeling, this finding may indicate that more nuanced joint prediction of poverty and malnutrition status may offer a promising direction of future work, which until now has been overlooked in the literature. We note as well that physical geography features contribute the most to predictive performance, underscoring the value of geographic targeting and the importance of good spatial data. Weather and vegetation data are relatively more important for sequential nowcasting associated with early warning tasks than with contemporaneous prediction.

The modest success of our independent and Mahalanobis random forest models signals that such methods and free, publicly available data can generate predictions of poverty and malnutrition prevalence that are comparable in accuracy to past published studies relying on deep or transfer learning based methods, proprietary data, or both, when predictions were assessed in aggregate and at country-specific levels. However, we found the performance of our model deteriorated considerably when predictions were assessed at the level of individual surveys in our nowcasting framework, as measured by *r*^2^, due largely to poor performance on initial, small sample size, surveys, alongside a less extreme drop in performance when assessing contemporaneous results at this scale. This shortcoming can likely be overcome through enhanced survey and data collection efforts, and as data availability continues to grow, the advantage that transfer learning methods have in overcoming sparse data problems may attenuate further. In contrast to our more variable performance measured by *r*^2^, predictive errors reflected in normalized root mean squared errors average just 10–25 percent of the range of the target prevalence rate of interest in both contemporaneous and sequential estimation, for each of the five malnutrition and poverty indicators we study, and at all levels of aggregation, signalling that big data and machine learning methods have real potential to inform development and humanitarian programming as data become more widely available and further methodologies are tested.

Despite promising initial results, statistical efforts towards the prediction of poverty and malnutrition status using RS data are far from complete, and for now, are unlikely to entirely replace traditional survey based methods and other approaches to develop actionable indicators for development and humanitarian agencies. Continued survey efforts will serve only to better facilitate the estimation of poverty and malnutrition status, and may broaden the range of statistical methods which can be applied to this task. As the viability and validation of these methods advance, consideration of the use cases for estimates generated by these methods requires greater attention, as different predictive methods may suit early warning tasks moreso than geographic targeting tasks, as was the case for our MRF model, and as discussed in more detail in [1]. Furthermore, ML-based estimates of otherwise-unobserved prevalence rates can serve as useful features themselves, for example in predicting the changing fiscal demands of social protection programs in response to major shocks, with appropriate correction for the attenuated variance that inevitably arises with any generated regressor.

## Supporting information

S1 Data (CSV)

S1 File Survey level predictive results and additional figures. This file contains an analysis of our individual survey level predictive results, displays additional spatial nowcast maps, displays market locations in relation to DHS EAs, and contains a few miscellaneous figures such as links to data sources and records of DHS survey sizes.
(PDF)
