# Supplementary material for: Multivariate random forest prediction of poverty and malnutrition prevalence
Source: PLoS One. 2021 Sep 8;16(9):e0255519. doi: 10.1371/journal.pone.0255519 (PMC8425567; doi:10.1371/journal.pone.0255519)

S1 Appendix: Predictive results indexed by survey

To examine how the quality of our predictions vary across individual surveys, we provide in Figs S1, S2, S3, S4, S5, full, out-of-sample, r^2 sampling distributions for prediction of DHS prevalence rates at the survey-specific level, for both sequential nowcasting and contemporaneous prediction and for both independent and Mahalanobis random forests. In Tables S1, S2, S3, S4, we present summary statistics which summarize these results.

Table S1. Summary statistics for sequential nowcasting with independent random forests with performance measures computed separately for each survey.

	Child Stunting	Child Wasting	Healthy Weight	Asset Poverty	Underwt Women
Mean r^2	0.00	-0.25	-0.38	-0.29	0.09
Median r^2	0.01	-0.03	-0.07	0.14	0.08
Std r^2	0.02	0.14	0.44	2.7	0.03
Mean NRMSE	0.21	0.15	0.16	0.26	0.17
Median NRMSE	0.21	0.16	0.16	0.23	0.18
Std NRMSE	0.00	0.00	0.00	0.01	0.00

Table S2. Summary statistics for sequential nowcasting with joint random forests with performance measures computed separately for each survey.

	Child Stunting	Child Wasting	Healthy Weight	Asset Poverty	Underwt Women
Mean r^2	0.01	-0.05	-0.17	-0.12	0.07
Median r^2	0.00	-0.02	-0.02	0.14	0.05
Std r^2	0.02	0.02	0.10	1.28	0.03
Mean NRMSE	0.21	0.14	0.15	0.26	0.17
Median NRMSE	0.21	0.15	0.16	0.23	0.18
Std NRMSE	0.00	0.00	0.00	0.01	0.00

Table S3. Summary statistics for contemporaneous prediction with independent random forests with performance measures computed separately for each survey.

	Child Stunting	Child Wasting	Healthy Weight	Asset Poverty	Underwt Women
Mean r^2	0.17	0.07	0.04	0.44	0.20
Median r^2	0.09	0.00	0.00	0.46	0.23
Std r^2	0.03	0.02	0.01	0.03	0.02
Mean NRMSE	0.21	0.18	0.19	0.20	0.19
Median NRMSE	0.22	0.20	0.21	0.21	0.19
Std NRMSE	0.00	0.00	0.00	0.00	0.00

Table S4. Summary statistics for Contemporaneous prediction with joint random forests, with performance measures computed separately for each survey.

	Child Stunting	Child Wasting	Healthy Weight	Asset Poverty	Underwt Women
Mean r^2	0.17	0.08	0.04	0.43	0.19
Median r^2	0.09	0.01	0.00	0.45	0.16
Std r^2	0.02	0.02	0.01	0.03	0.02
Mean NRMSE	0.21	0.18	0.19	0.20	0.19
Median NRMSE	0.23	0.20	0.21	0.21	0.19
Std NRMSE	0.00	0.00	0.00	0.00	0.00

.

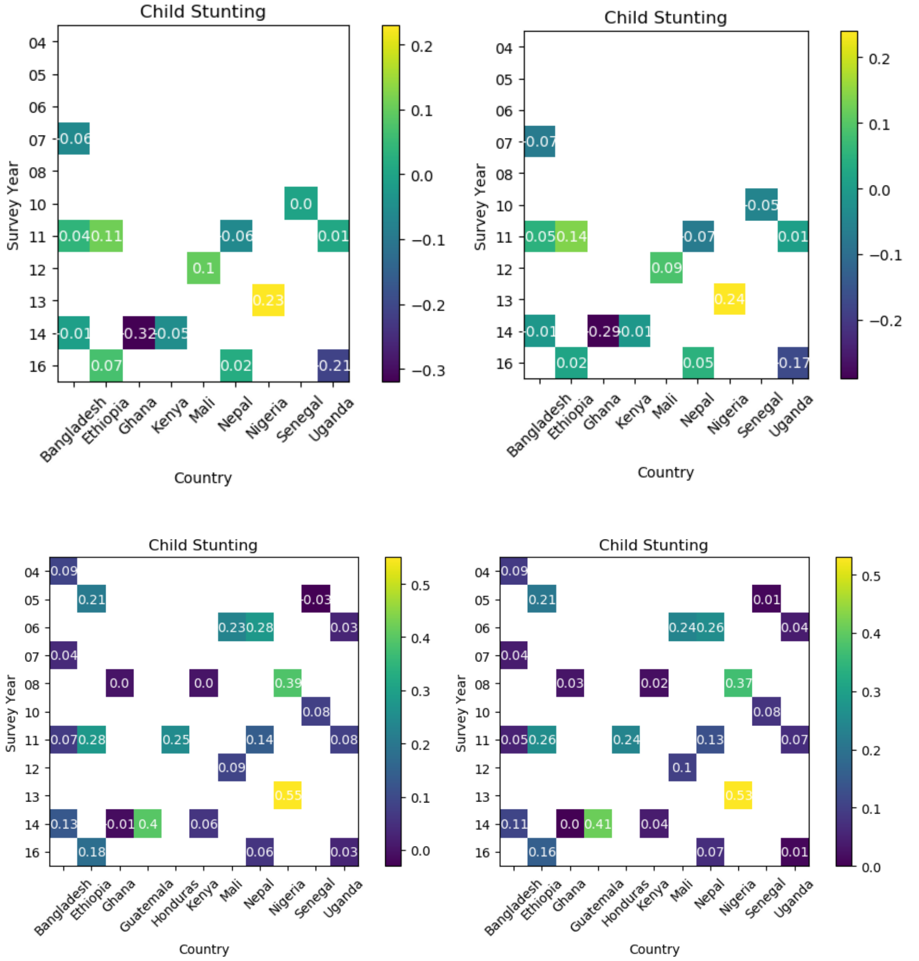

Fig S1. r^2 **for Child Stunting prediction, indexed by year, country.** Top row: sequential, bottom row: contemporaneous. Left column: Independent RF, Right column: MRF

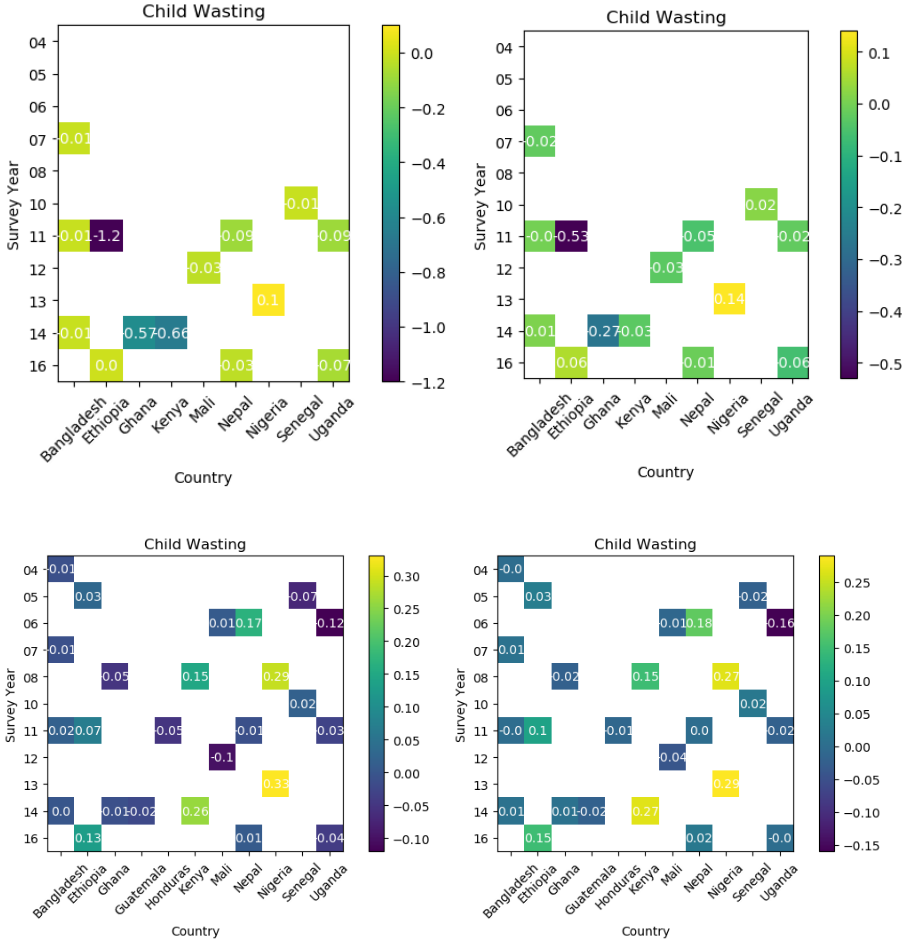

Fig S2. r^2 **for Child Wasting prediction, indexed by year, country.** Top row: sequential, bottom row: contemporaneous. Left column: Independent RF, Right column: MRF

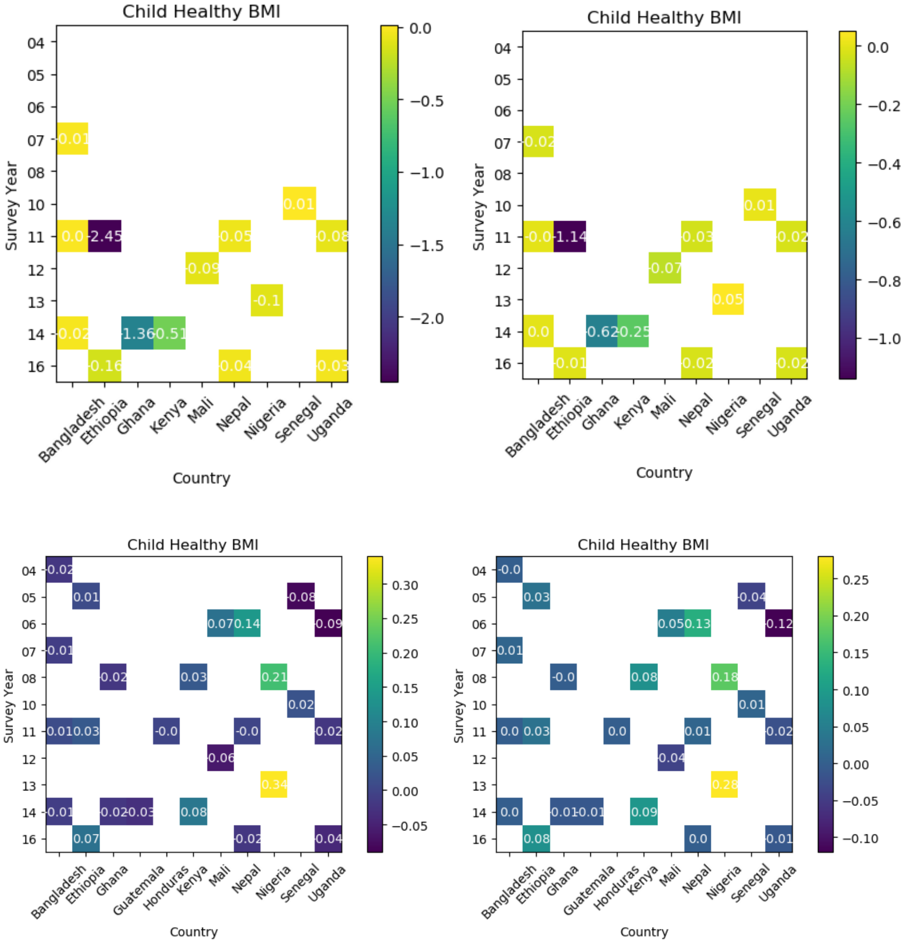

Fig S3. r^2 for Healthy Weight Children prediction, indexed by year, country. Top row: sequential, bottom row: contemporaneous. Left column: Independent RF, Right column: MRF

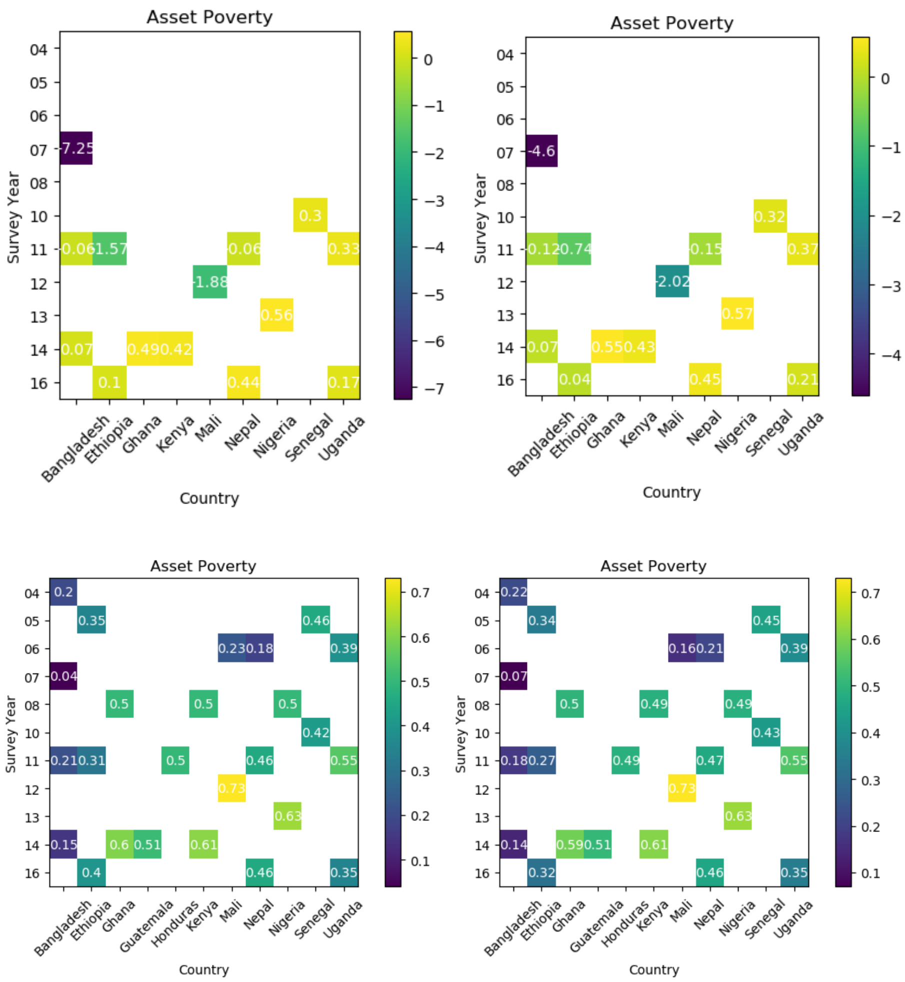

Fig S4. r^2 **for Asset Poverty prediction, indexed by year, country.** Top row: sequential, bottom row: contemporaneous. Left column: Independent RF, Right column: MRF

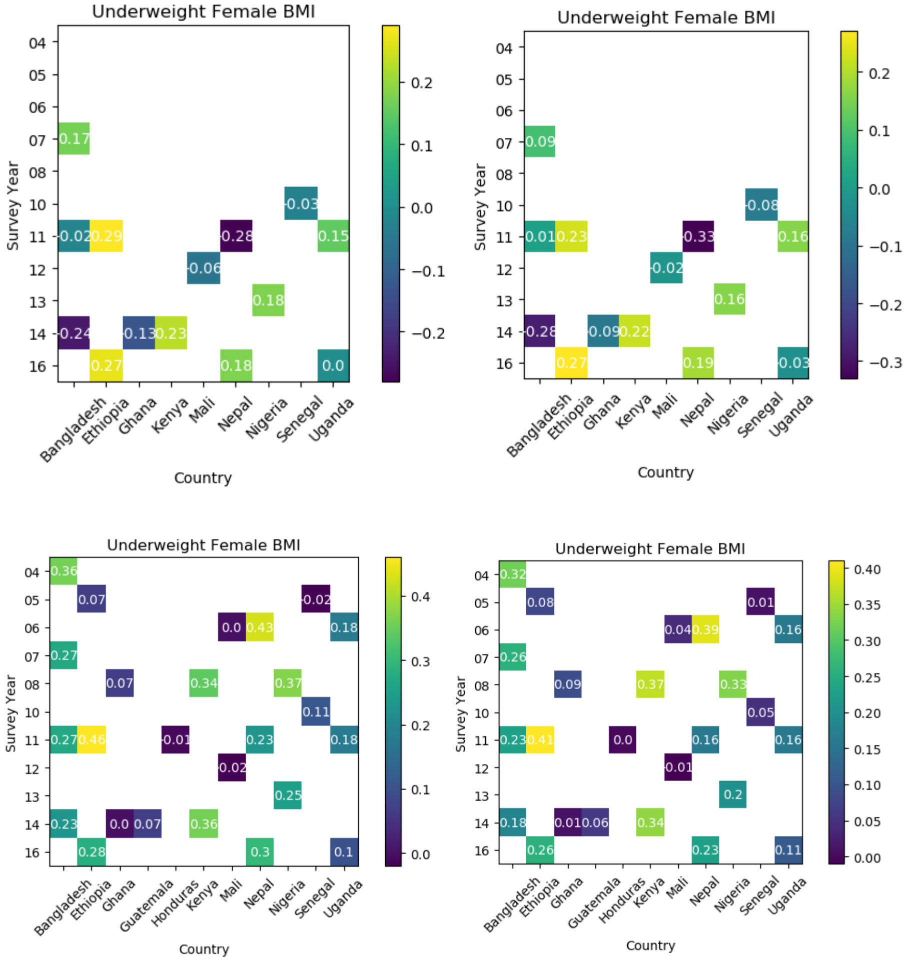

Fig S5. r^2 for Underweight Female BMI prediction, indexed by year, country. Top row: sequential, bottom row: contemporaneous. Left column: Independent RF, Right column: MRF

Relative to our aggregate results shown in Tables 1,3, we immediately notice a significant drop in average r^2 for sequential nowcasting of all prevalence rates, alongside a less extreme but still significant decrease in r^2 for contemporaneous prediction, for both joint and independent random forest models. Surprisingly, we find that our NRMSEs stay qualitatively similar to our aggregate results, with errors remaining bound by 10 to 25 percent of an indicators given range, indicating our model can still produce relatively reliable prevalence rate predictions for the purpose of policy informance at survey-level scale, but that these predictions fail to explain the underlying variability in prevalence rates over small spatial domains as a function of input features. This reduction in performance is accompanied by high variability in out-of-sample r^2, and, particularly in the case of sequential nowcasting, the emergence of a heavy tail of poor performance. This finding is best emphasized in our nowcasting of asset poverty prevalence in Bangladesh 2007, Ethiopia 2011, and Mali 2012 (Fig S4). In contrast, we see surveys with relatively high performance, such as Nigeria, 13. Noting these catastrophic results coincide in all cases with the earliest possible testing years in relatively small sample size countries, where training and testing sets can be on the order of as few as 300-400 samples (see Table S6 for exact survey sizes), it

seems quite plausible these results are caused by small sample sizes which are insufficient to train random forest models capable of effectively discriminating poverty and malnutrition prevalence. In support of this hypothesis, we display in Fig S6 r^2 and NRMSE as a function of survey size, and note a clear trend moving from small to large scale surveys.

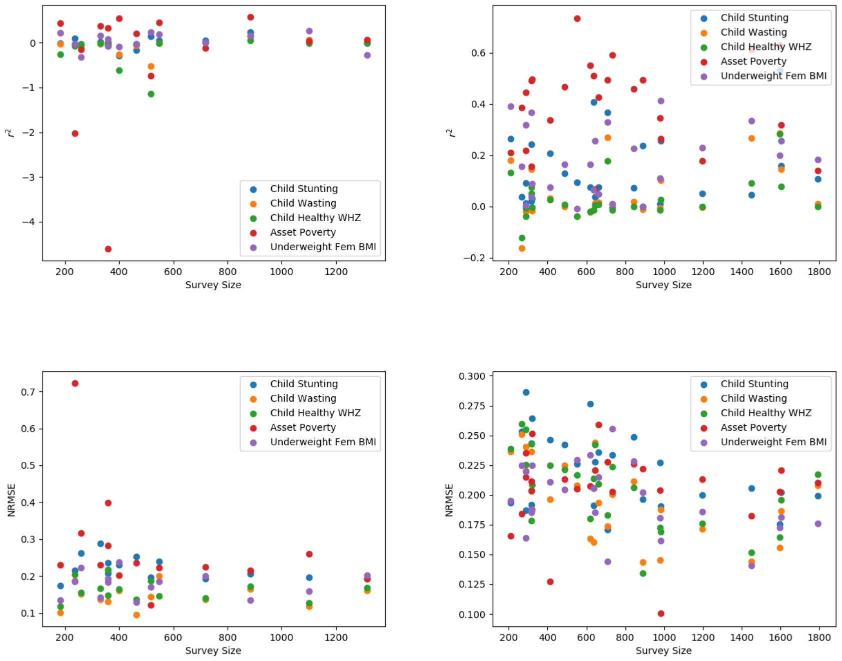

Fig S6. Out-of-sample r^2 and NRMSE for poverty and malnutrition prevalence prediction using MRFs as function of training size. Left Column: Sequential Right: Contemporaneous

While these findings pose a strong motivation for continued collection of poverty and malnutrition surveys to facilitate future remote sensing efforts, it also indicates that the results of our model must be taken with a grain of salt, particularly when generating nowcasts over small spatial scales from minimal training data, and indicate a relative strength of transfer learning approaches which are less reliant on sample size. Nevertheless, our positive aggregate results, the existence of some positive results for individual surveys, and generally small NRMSE across these two predictive tasks, seems to indicate our methodology can still be used to generate reasonable first order assessments of poverty and malnutrition prevalence, particularly in countries with higher survey engagement.

S2 Appendix: Market and enumeration area locations

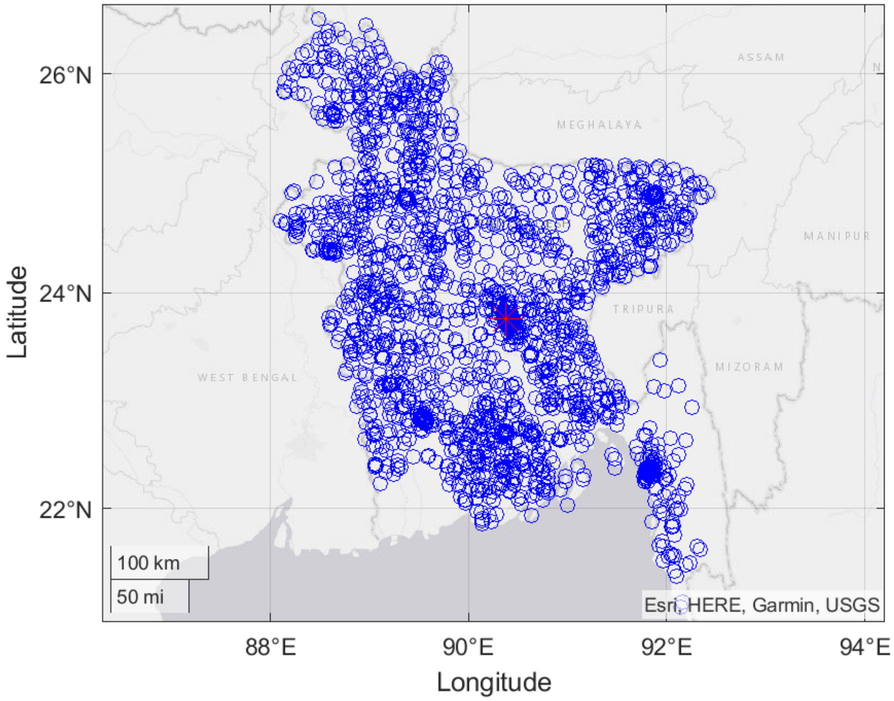

Fig S7. Overview of Enumeration Areas and Markets for Bangladesh. Blue dots represent EA's, with all EA's used across all surveys pictured in this figure. Red stars mark market locations.

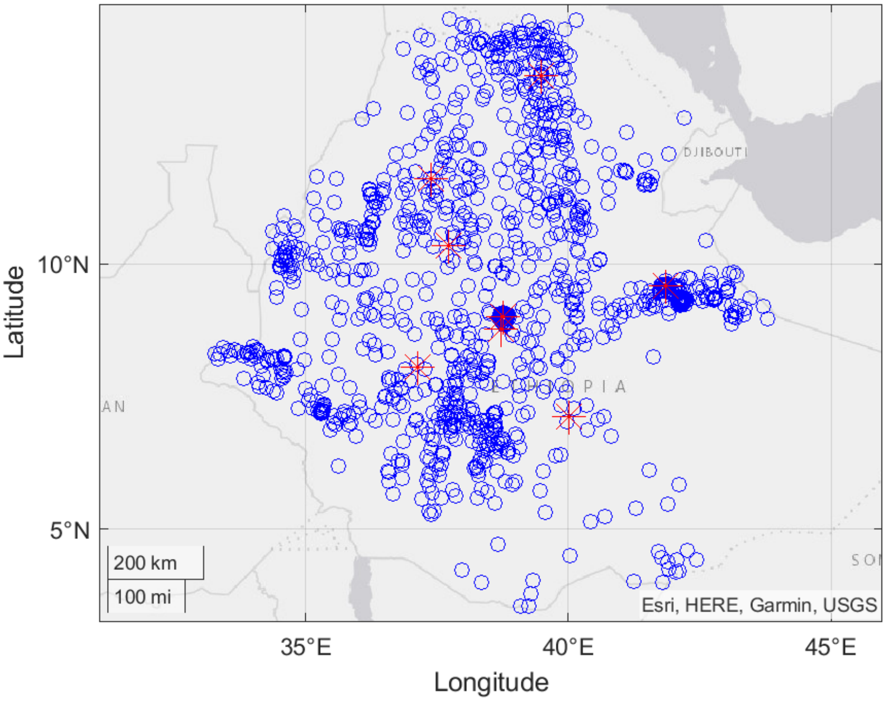

Fig S8. Overview of Enumeration Areas and Markets for Ethiopia. Blue dots represent EA's, with all EA's used across all surveys pictured in this figure. Red stars mark market locations.

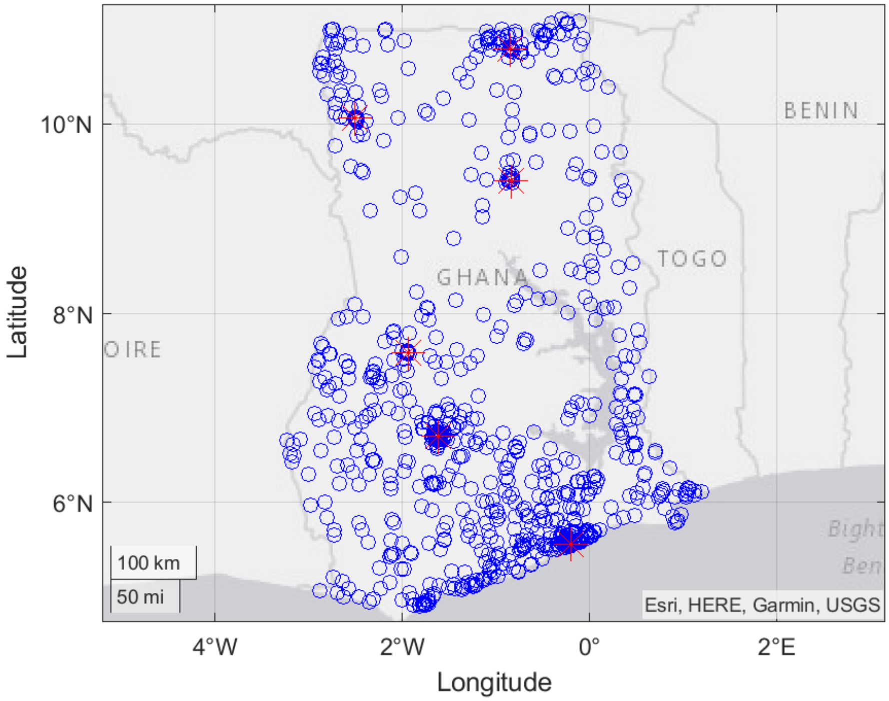

Fig S9. Overview of Enumeration Areas and Markets for Ghana. Blue dots represent EA's, with all EA's used across all surveys pictured in this figure. Red stars mark market locations.

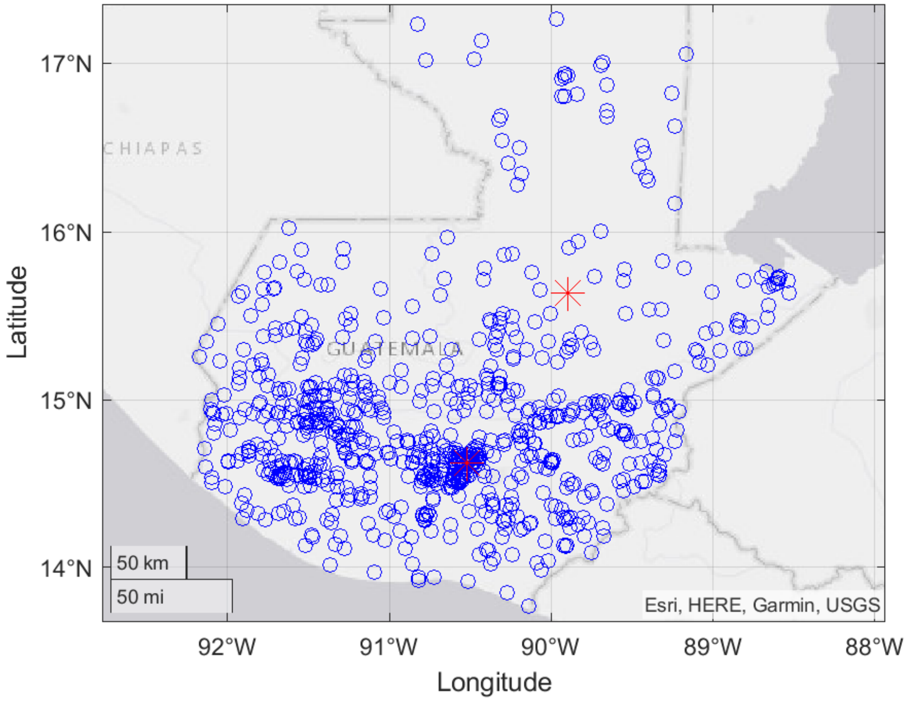

Fig S10. Overview of Enumeration Areas and Markets for Guatemala. Blue dots represent EA's, with all EA's used across all surveys pictured in this figure. Red stars mark market locations.

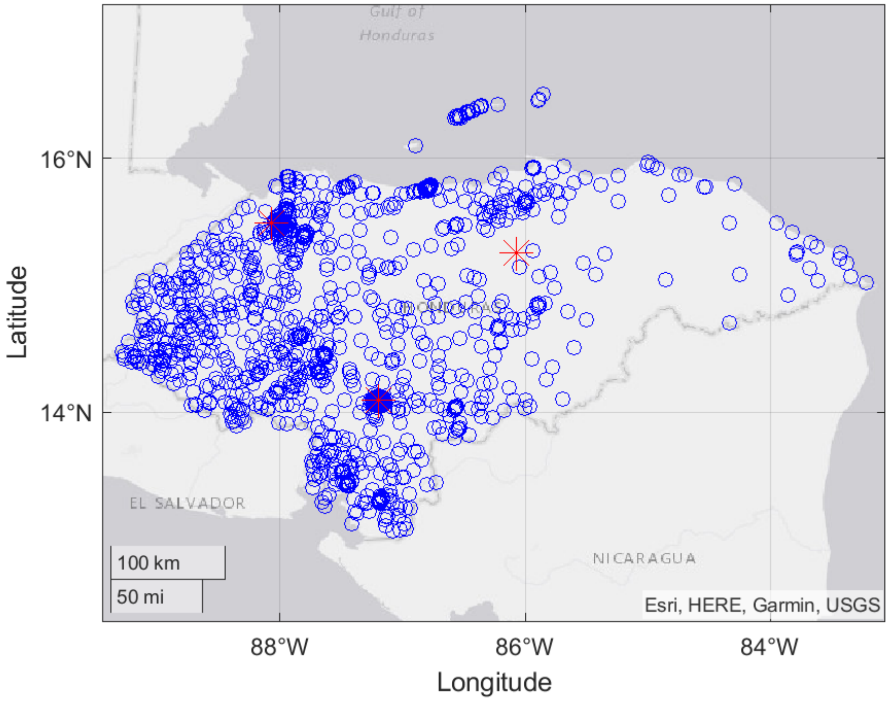

Fig S11. Overview of Enumeration Areas and Markets for Honduras. Blue dots represent EA's, with all EA's used across all surveys pictured in this figure. Red stars mark market locations.

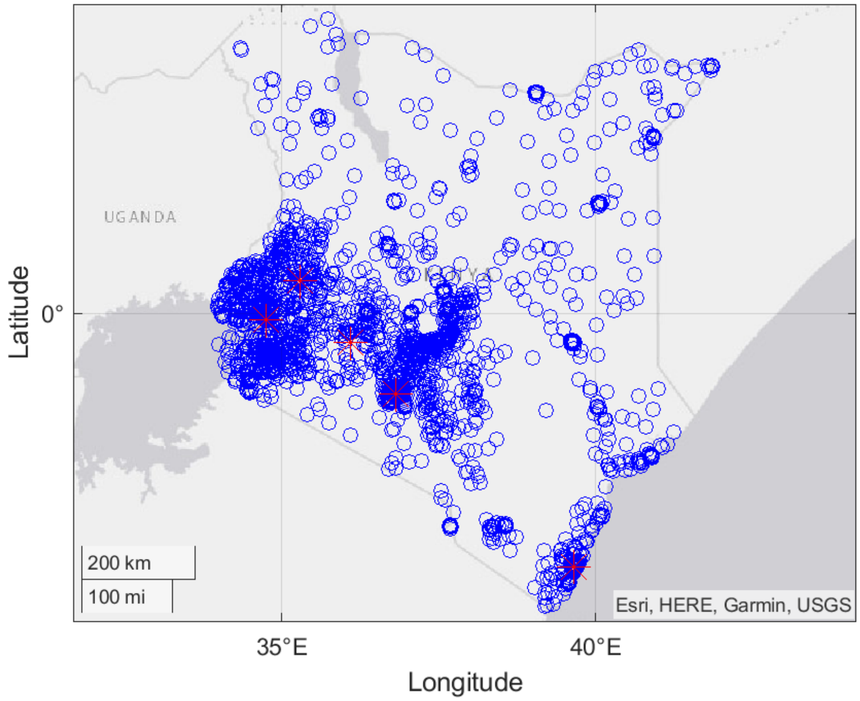

Fig S12. Overview of Enumeration Areas and Markets for Kenya. Blue dots represent EA's, with all EA's used across all surveys pictured in this figure. Red stars mark market locations.

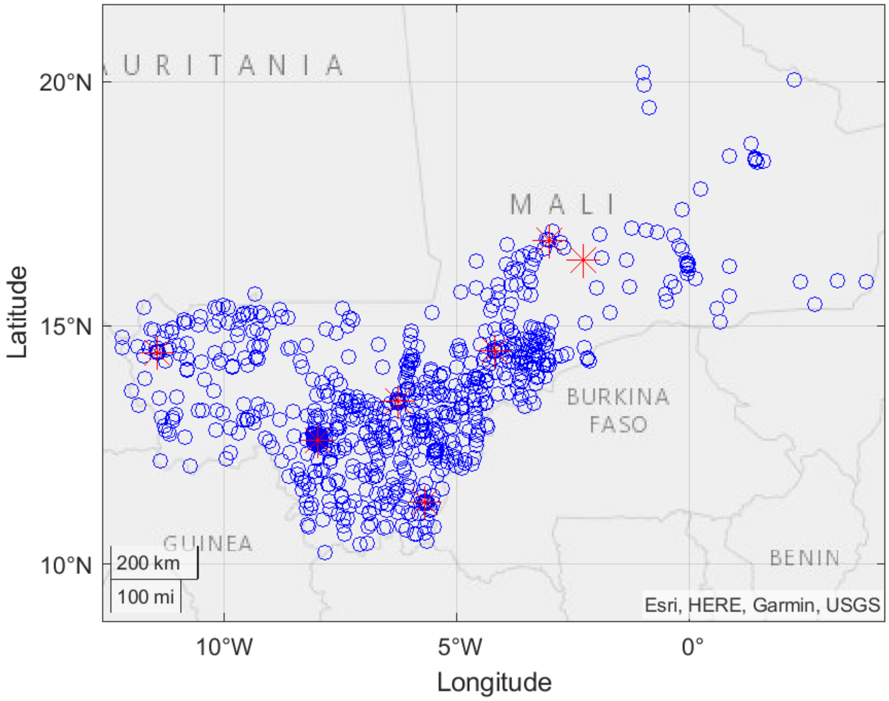

Fig S13. Overview of Enumeration Areas and Markets for Mali. Blue dots represent EA's, with all EA's used across all surveys pictured in this figure. Red stars mark market locations.

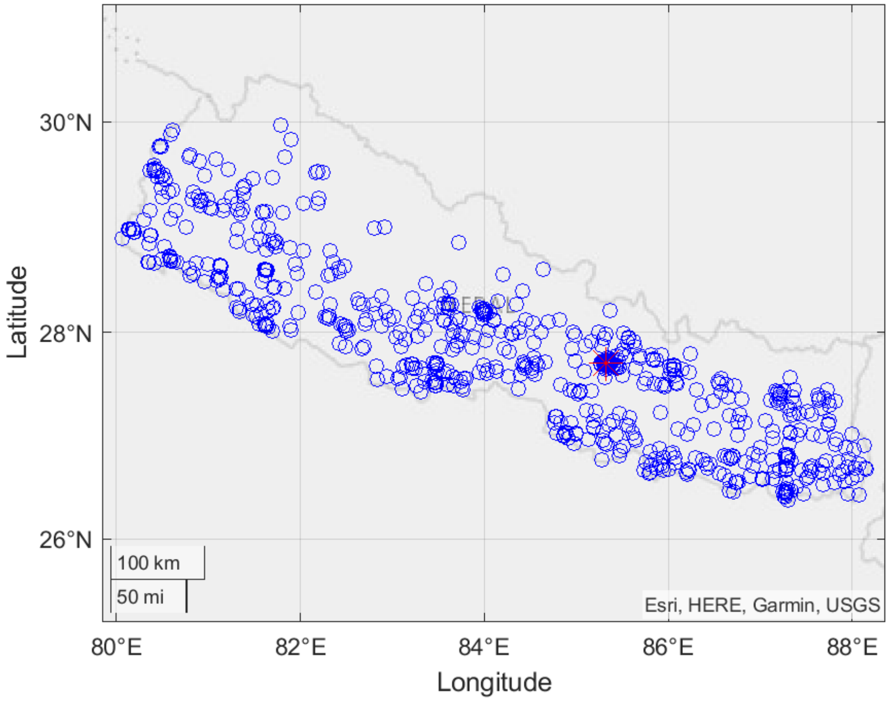

Fig S14. Overview of Enumeration Areas and Markets for Nepal. Blue dots represent EA's, with all EA's used across all surveys pictured in this figure. Red stars mark market locations.

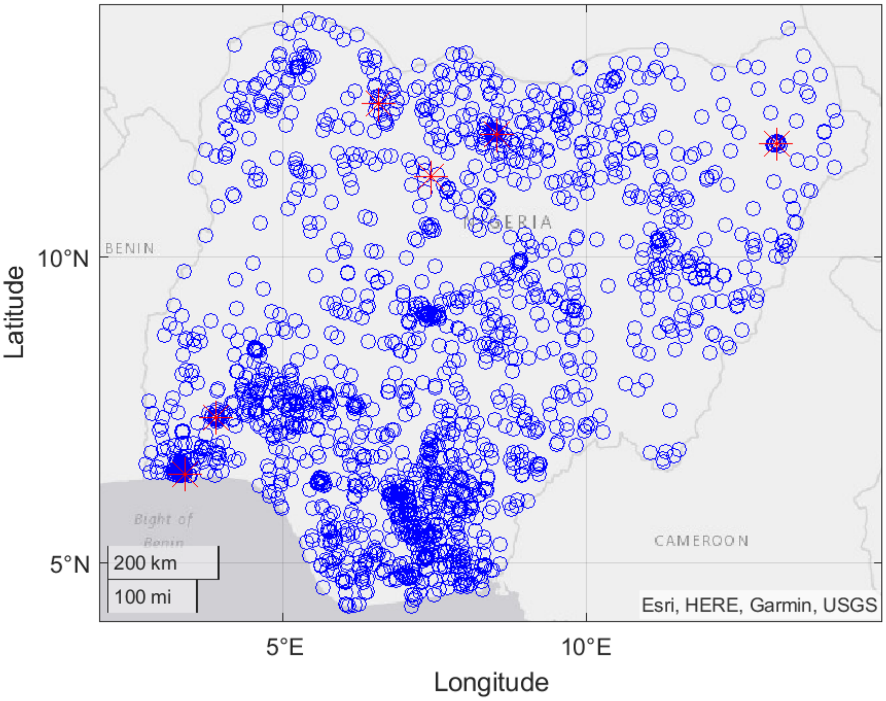

Fig S15. Overview of Enumeration Areas and Markets for Nigeria. Blue dots represent EA's, with all EA's used across all surveys pictured in this figure. Red stars mark market locations.

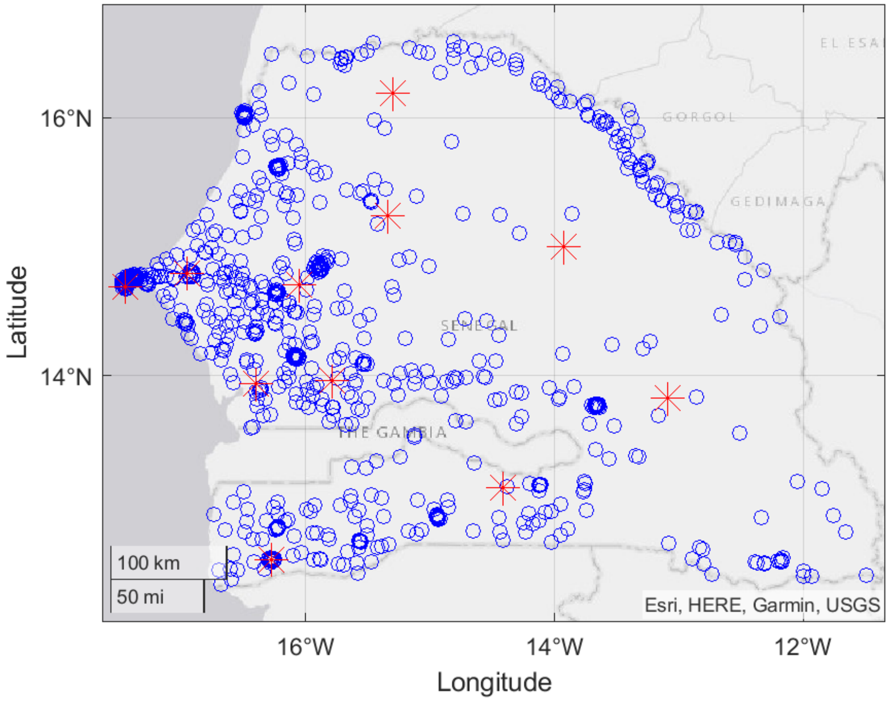

Fig S16. Overview of Enumeration Areas and Markets for Senegal. Blue dots represent EA's, with all EA's used across all surveys pictured in this figure. Red stars mark market locations.

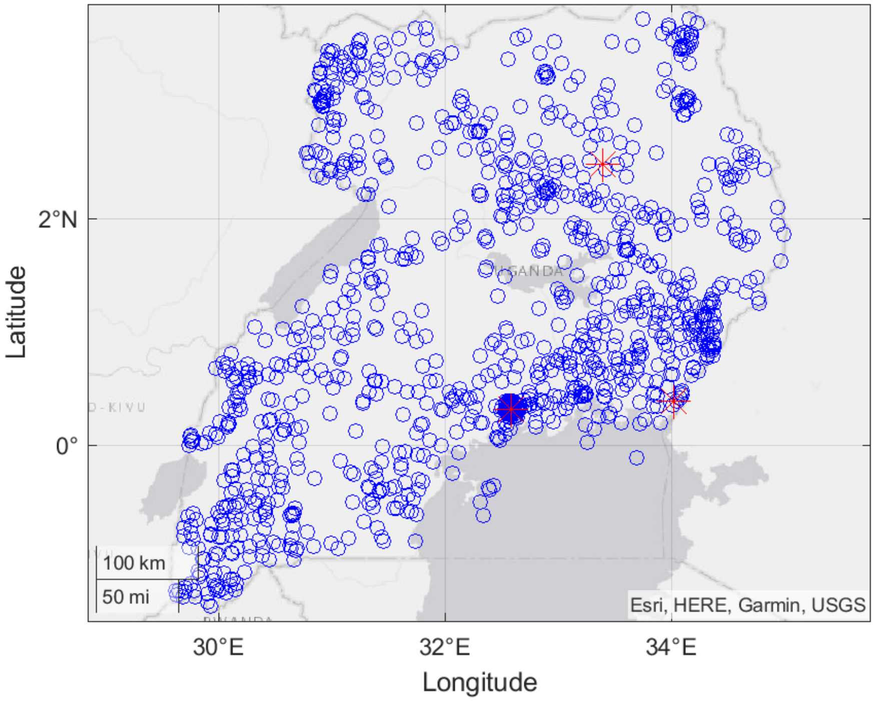

Fig S17. Overview of Enumeration Areas and Markets for Uganda. Blue dots represent EA's, with all EA's used across all surveys pictured in this figure. Red stars mark market locations.

S3 Appendix: Additional spatial nowcast maps

Note that areas within these figures lacking predictions correspond to areas where our our full set of RS data is unavailable.

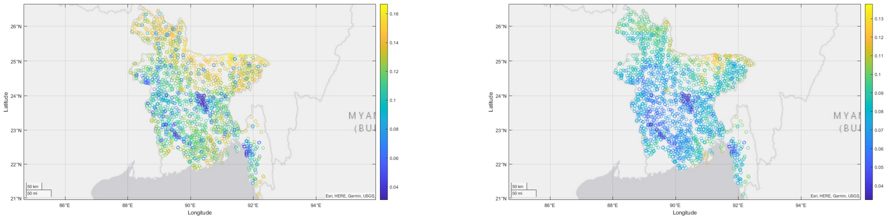

Fig S18. Nowcast of asset poverty prevalence for Bangladesh, 2014. (Left): Mean prediction (Right): Standard deviation of predictions across trees.

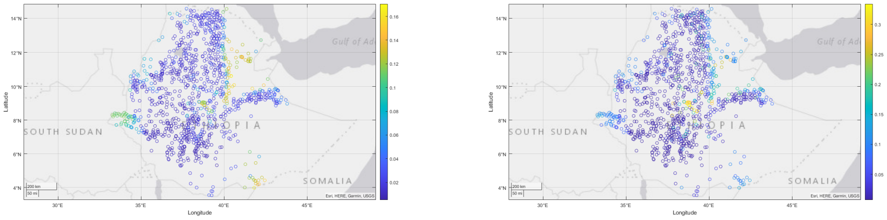

Fig S19. Nowcast of asset poverty prevalence for Ethiopia, 2016. (Left): Mean prediction (Right): Standard deviation of predictions across trees.

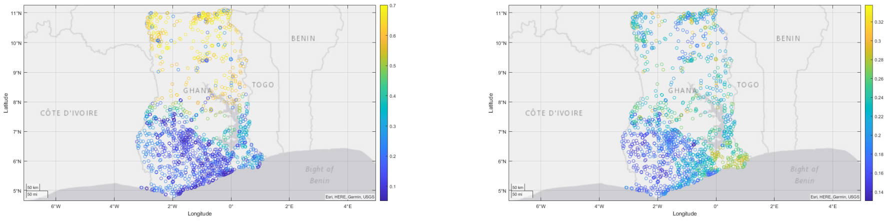

Fig S20. Nowcast of asset poverty prevalence for Ghana, 2014. (Left): Mean prediction (Right): Standard deviation of predictions across trees.

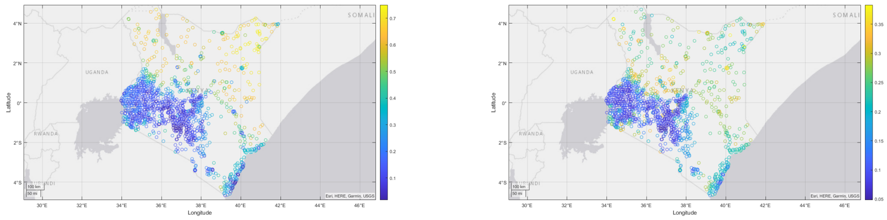

Fig S21. Nowcast of asset poverty prevalence for Kenya, 2014. (Left): Mean prediction (Right): Standard deviation of predictions across trees.

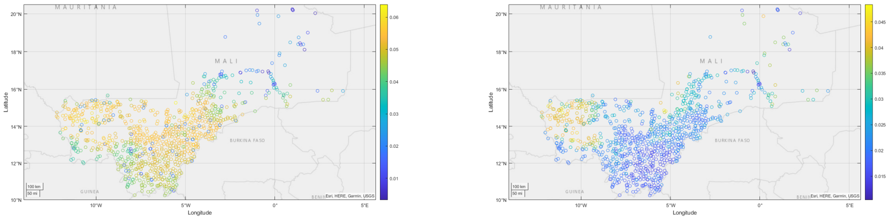

Fig S22. Nowcast of asset poverty prevalence for Mali, 2012. (Left): Mean prediction (Right): Standard deviation of predictions across trees.

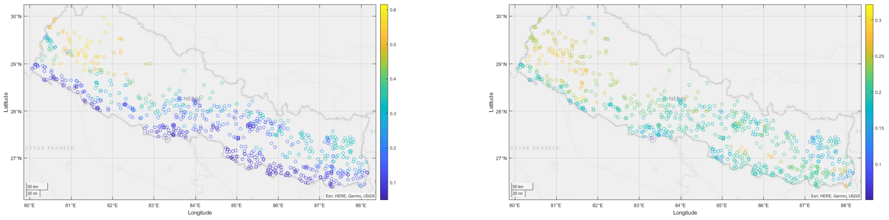

Fig S23. Nowcast of asset poverty prevalence for Nepal, 2016. (Left): Mean prediction (Right): Standard deviation of predictions across trees.

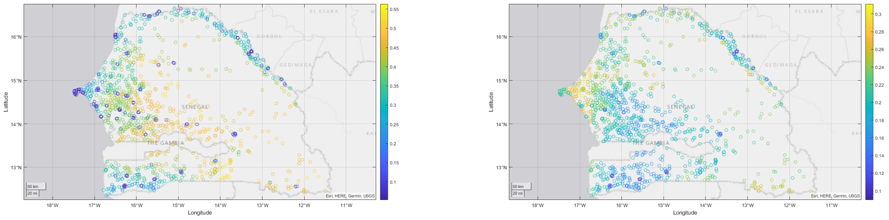

Fig S24. Nowcast of asset poverty prevalence for Senegal, 2010. (Left): Mean prediction (Right): Standard deviation of predictions across trees.

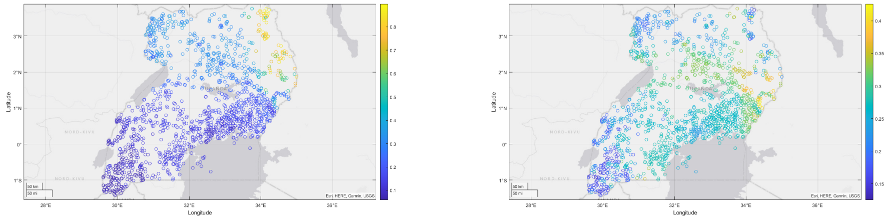

Fig S25. Nowcast of asset poverty prevalence for Uganda, 2016. (Left): Mean prediction (Right): Standard deviation of predictions across trees.

S4 Appendix: Additional tables and figures

Table S5. Summary of data sources and corresponding download links.

Data	Link
ARENA [23]	`https://dataverse.harvard.edu/dataset.xhtml?persistentId=doi:10.7910/DVN/OQIPRW`
DHS [24]	`https://dhsprogram.com/data/available-datasets.cfm`
CHRPS [55]	`https://www.chc.ucsb.edu/data`
LST [49]	Data are not yet available for public use. Interested parties can contact Leiqiu Hu for details on when the data will be available. Please see `https://huleiqiu.wordpress.com/data-2/`
SIF [27]	`https://www.yingsun.info/datasets-and-tools`
FAO [31]	`https://fpma.apps.fao.org/giews/food-prices/tool/public/#/home`
UCDP [62] [63]	`https://ucdp.uu.se/downloads/index.html#ged_global`
Processed data	`http://barrett.dyson.cornell.edu/files/research/data.csv`

Table S6. Number of DHS enumeration areas used for training and prediction, by country and survey year.

	2004	2005	2006	2007	2008	2010	2011	2012	2013	2014	2016
Bangladesh	361	0	0	361	0	0	600	0	0	600	0
Ethiopia	0	523	0	0	0	0	596	0	0	0	640
Ghana	0	0	0	0	402	0	0	0	0	418	0
Guatemala	0	0	0	0	0	0	0	0	0	802	0
Honduras	0	0	0	0	0	0	1135	0	0	0	0
Kenya	0	0	0	0	397	0	0	0	0	1592	0
Mali	0	0	406	0	0	0	0	407	0	0	0
Nepal	0	0	260	0	0	0	287	0	0	0	370
Nigeria	0	0	0	0	886	0	0	0	895	0	0
Senegal	0	368	0	0	0	388	0	0	0	0	0
Uganda	0	0	363	0	0	0	393	0	0	0	688

Note that although DHS surveys also exist in 2003 for Ghana, Kenya, and Nigeria, these surveys are omitted from our analysis due to inavailability of SIF data for these years. The 2012 and 2014 Senegal DHS surveys are omitted due to missing Underweight Female BMI data.

Table S7. Summary of open access food price data from FAO

Country	No. of food types	No. of markets	Price Type	First available period
Bangladesh	5	1	Retail	7/1998
Ethiopia	5	9	Wholesale	1/2000
Ghana	8	6	Wholesale	1/2006
Guatemala	7	2	Wholesale	1/2000
Honduras	3	3	Wholesale	‘1/2000
Kenya	2	5	Wholesale	1/2006
Mali	4	7	Wholesale	1/2005
Nepal	2	1	Retail	1/2005
Nigeria	6	6	Wholesale	8/2003
Senegal	3	11	Retail	1/2007
Uganda	5	3	Wholesale	1/2006

Fig S26. Each dot represents the expected change in prevalence rate when moving between adjacent survey rounds within each country. Note that countries with only a single survey round are omitted.

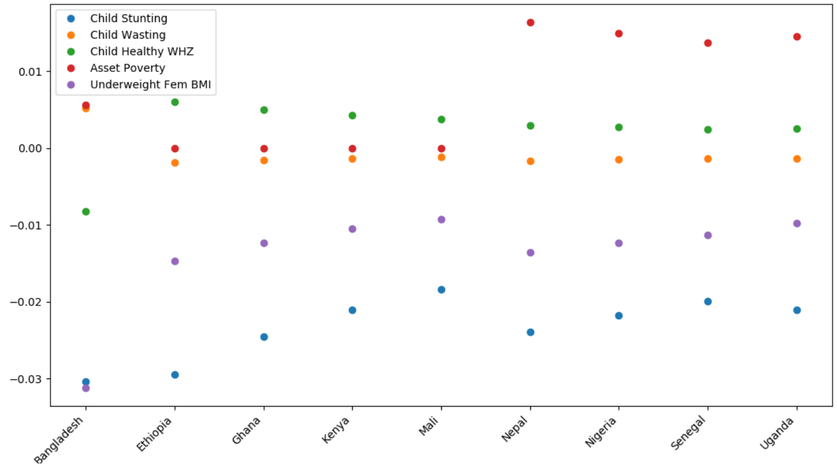

Supplement: S1 File — This file contains an analysis of our individual survey level predictive results, displays additional spatial nowcast maps, displays market locations in relation to DHS EAs, and contains a few miscellaneous figures such as links to data sources and records of DHS survey sizes. (PDF) [file pone.0255519.s002.pdf]
